# A Phenotypic and Genotypic Analysis of the Antimicrobial Potential of Cultivable *Streptomyces* Isolated from Cave Moonmilk Deposits

**DOI:** 10.3389/fmicb.2016.01455

**Published:** 2016-09-21

**Authors:** Marta Maciejewska, Delphine Adam, Loïc Martinet, Aymeric Naômé, Magdalena Całusińska, Philippe Delfosse, Monique Carnol, Hazel A. Barton, Marie-Pierre Hayette, Nicolas Smargiasso, Edwin De Pauw, Marc Hanikenne, Denis Baurain, Sébastien Rigali

**Affiliations:** ^1^InBioS – Centre for Protein Engineering, Institut de Chimie B6a, University of LiègeLiège, Belgium; ^2^Environmental Research and Innovation Department, Luxembourg Institute of Science and TechnologyBelvaux, Luxembourg; ^3^InBioS – Plant and Microbial Ecology, Botany B22, University of LiègeLiège, Belgium; ^4^Department of Biology, University of Akron, AkronOH, USA; ^5^Department of Clinical Microbiology, University Hospital of LiègeLiège, Belgium; ^6^MolSys Research Unit, Mass Spectrometry Laboratory, University of LiègeLiège, Belgium; ^7^InBioS – Functional Genomics and Plant Molecular Imaging, University of LiègeLiège, Belgium; ^8^PhytoSYSTEMS, University of LiègeLiège, Belgium; ^9^InBioS – Eukaryotic Phylogenomics, University of LiègeLiège, Belgium

**Keywords:** geomicrobiology, secondary metabolism, MLSA phylogeny, cryptic antibiotics, genome mining

## Abstract

Moonmilk speleothems of limestone caves host a rich microbiome, among which Actinobacteria represent one of the most abundant phyla. Ancient medical texts reported that moonmilk had therapeutical properties, thereby suggesting that its filamentous endemic actinobacterial population might be a source of natural products useful in human treatment. In this work, a screening approach was undertaken in order to isolate cultivable Actinobacteria from moonmilk of the Grotte des Collemboles in Belgium, to evaluate their taxonomic profile, and to assess their potential in biosynthesis of antimicrobials. Phylogenetic analysis revealed that all 78 isolates were exclusively affiliated to the genus *Streptomyces* and clustered into 31 distinct phylotypes displaying various pigmentation patterns and morphological features. Phylotype representatives were tested for antibacterial and antifungal activities and their genomes were mined for secondary metabolite biosynthetic genes coding for non-ribosomal peptide synthetases (NRPSs), and polyketide synthases (PKS). The moonmilk *Streptomyces* collection was found to display strong inhibitory activities against a wide range of reference organisms, as 94, 71, and 94% of the isolates inhibited or impaired the growth of Gram-positive, Gram-negative bacteria, and fungi, respectively. Interestingly, 90% of the cave strains induced strong growth suppression against the multi-drug resistant *Rasamsonia argillacea*, a causative agent of invasive mycosis in cystic fibrosis and chronic granulomatous diseases. No correlation was observed between the global antimicrobial activity of an individual strain and the number of NRPS and PKS genes predicted in its genome, suggesting that approaches for awakening cryptic metabolites biosynthesis should be applied to isolates with no antimicrobial phenotype. Overall, our work supports the common belief that moonmilk might effectively treat various infectious diseases thanks to the presence of a highly diverse population of prolific antimicrobial producing *Streptomyces*, and thus may indeed constitute a promising reservoir of potentially novel active natural compounds.

## Introduction

Members of the phylum Actinobacteria can be found in all kinds of extreme environments (Saiz-Jimenez, 1999; Bull, 2010; Prieto-Davó et al., 2013; Zhang et al., 2014; Mohammadipanah and Wink, 2015; Shivlata and Satyanarayana, 2015). Their successful survival in severe conditions suggests broad adaptive abilities that might be directly related to their very diverse and specialized (secondary) metabolism. Natural small molecules, collectively termed the parvome (Davies and Ryan, 2012), apart from having essential ecological functions, possess a wide range of bioactivities, which are applicable for agro-industrial purposes and human/animal therapy (Hopwood, 2007). Since the metabolome of soil-dwelling Actinobacteria, especially of the members of the genus *Streptomyces*, has been widely exploited, leading to the multiple re-isolation of already known bioactive compounds, the attention has been refocused toward unexplored and extreme environments, which can potentially be a source of novel species and consequently of novel molecules of interest (Cheeptham et al., 2013; Claverías et al., 2015; Mohammadipanah and Wink, 2015; Liao et al., 2016).

The geological isolation of caves from surface processes makes them a unique niche, not only to study microbial interactions and adaptations to extreme oligotrophy, but also to screen for potentially novel bioactive compounds. Although, the most common Actinobacteria reported from caves belong to *Pseudonocardiaceae* and *Nocardiaceae* families (Stomeo et al., 2008; Porca et al., 2012; Quintana et al., 2013; Riquelme et al., 2015), many investigations have identified cultivable members of the genus *Streptomyces*, which are the most prolific antimicrobial producers (Cañaveras et al., 1999; Groth et al., 1999; Cheeptham et al., 2013; Nimaichand et al., 2015; Axenov-Gribanov et al., 2016). The presence of Actinobacteria as dominant members of microbial ecosystems in caves is puzzling, as these bacteria, particularly *Streptomyces* are often presented as major protagonists in the recycling of the residual plant biomass in nutrient-rich soil environments (Hodgson, 2000). Nonetheless, >99% of allochtonous carbon entering caves, primarily with drip water, contains soil-derived dissolved organic carbon in the form of partially degraded plant and fungal polymers, which can be catabolized by the enzymatic arsenals of *Streptomyces* (Saiz-Jimenez and Hermosin, 1999; Simon et al., 2007; Barton, 2015). Additionally, highly prolific and diversified secondary metabolism could be a driving force in the dominance of these species in oligotrophic environments, through which they could shape microbiomes thanks to their specialized metabolites, such as metal-chelators for acquiring trace metals, and antibiotics to prevent nutrient-exclusion by competitive species (Bhullar et al., 2012). Antibiotics do not exclusively prevent microbial growth, but are also known to act as inter-/intraspecies communication (Sengupta et al., 2013), and as cues triggering adaptations, such as motility or biofilm formation (Linares et al., 2006), or can be used as alternative carbon and/or nitrogen sources (Dantas et al., 2008). Consequently, small bioactive molecules expressed under nutrient-starved conditions, could be used as weapons, as signals, or as nutrient sources.

Moonmilk is a comparatively rare speleothem in cave environments, where it forms as a thick calcite paste (similar in consistency to toothpaste) up to 10s-of-centimeters in thickness in passageways that receive significant airflow (Hill and Forti, 1997; Borsato et al., 2000). The exact process of moonmilk formation remains in debate; however, the consistency, the high abundance of filamentous bacterial cells and members of the Actinobacteria has led researchers to suggest a biogenic origin (Cañaveras et al., 2006; Rooney et al., 2010; Portillo and Gonzalez, 2011). Despite its relative scarcity in caves, moonmilk has had long scientific interest due to its historical use as a medical treatment. Interestingly, moonmilk was used in human and animal therapy since the Middle Ages (Reinbacher, 1994). Its curative properties could be associated with the presence of the numerous filamentous Actinobacteria, particularly *Streptomyces*, presumably producing bioactive molecules. Indeed, isolation of *Streptomyces* with antimicrobial and antifungal activities has been reported for moonmilk from the Bolshaya Oreshnaya Cave in Siberia (Axenov-Gribanov et al., 2016). The identification of potential novel compounds with broad-spectrum activities from *Streptomyces* found in this karstic secondary deposit supports the idea of moonmilk being a great target for bioactivity screening, although to date very few studies have examined cultivable moonmilk microbiome and the diversity of its metabolome as a possible reservoir of novel compounds. The fact that caves are unique and still highly under-explored environment increase the chances of finding novel organisms and consequently novel bioactive compounds that might be useful in the context of the global health problem of antibiotic resistance (Bush et al., 2011; Laxminarayan et al., 2013; Berendonk et al., 2015; Frère and Rigali, 2016).

In this work, we report the isolation and phylogenetic analysis of a collection of novel *Streptomyces* isolates from moonmilk deposits, and assess their potential as producers of compounds with antimicrobial and antifungal properties through *in vitro* screening and genome mining approaches.

## Materials and Methods

### Site Description and Sampling

**FIGURE 1:**
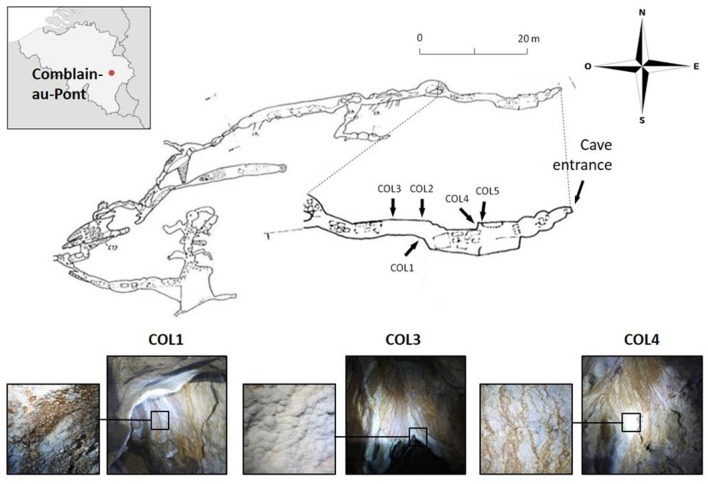
**Cave map of the Grotte des Collemboles and visualization of the moonmilk deposit sampling points.** Location of the Grotte des Collemboles (Springtails’ Cave) in Comblain-au-Pont (Liège, Belgium) and cave map with general view and close up of the moonmilk deposits from the different collection points (COL).

The cave called Grotte des Collemboles (Springtails’ Cave) located in Comblain-au-Pont in Belgium is a shallow (<20 m), ∼70 m long fissure cave, formed in the upper Viséan limestone (**Figure 1**), with an average annual temperature of ∼11.5°C. Due to cave protection policies and location on private property, specific location details and access information is available to other researchers upon request. Within the cave, white to brown-orange (presumably from iron-oxide precipitates) moonmilk deposits are found on the walls within the first 20 m of the cave in the first narrow chamber located at the entrance of the cave as well as in the narrow passages leading deeper into the cave (**Figure 1**). Samples used in this work were aseptically collected in January 2012 from three moonmilk deposits (**Figure 1**). Soft moonmilk speleothem was scratched with sterile scalpels from the wall in the first chamber, adjacent to the cave entrance (COL4) and the walls in a narrow passage after the first chamber (COL1, COL3; **Figure 1**). Samples were collected into falcon tubes, transferred to the laboratory, freeze-dried on a VirTis Benchtop SLC Lyophilizer (SP Scientific, Warminster, PA, USA) and stored at -20°C.

### Isolation of Cultivable Actinobacterial Species

Selective isolation of *Streptomyces* species from moonmilk was carried out by a serial dilution method as described previously (Maciejewska et al., 2015). 250 mg of lyophilized moonmilk sample from each collection point was suspended in 0.25X strength Ringer’s solution supplemented with 0.001% Tween 80. Resulting moonmilk suspensions were serially diluted in PBS and inoculated in duplicates on ISP media (Shirling and Gottlieb, 1966), starch nitrate (SN) medium (Gauze’s medium No.1; Waksman and Lechevalier, 1961), B-4 agar (Boquet et al., 1973), and minimal medium (Kieser et al., 2000) with 1% chitin (MMch). Isolation media were supplemented with nalidixic acid (75 μg/ml) and nystatin (50 μg/ml) to suppress the growth of Gram-negative bacteria and fungi, respectively. After 1 month of incubation at 17°C, colony forming units (CFUs) were enumerated and 129 isolates were selected. After two rounds of subcultivation, 78 isolates were recovered as purified strains and subsequently preserved both on ISP2 slopes at 4°C and as 25% glycerol mycelium stock at -20°C.

### DNA Extraction, Genome Sequencing, and Gene Selection from Moonmilk-Derived Isolates

In order to screen for the genes of interest, which would enable to identify moonmilk derived isolates, to perform phylogenetic analysis, as well as to investigate strains antimicrobial properties, *de novo* genome sequencing was carried out. DNA from purified strains was extracted with GenElute Bacterial Genomic DNA Kit (Sigma-Aldrich, St. Louis, MO, USA) according to manufacturer’s instructions from liquid LB (Luria-Bertani; Difco, BD, Franklin Lakes, NJ, USA) cultures incubated at 28°C. The genomic libraries of moonmilk isolates were constructed using Nextera XT kit (Illumina, Inc., San Diego, CA, USA). Library concentrations and mean fragment lengths were measured by Qubit fluorometer (Invitrogen, Grand Island, NY, USA) and Agilent Bioanalyzer (Agilent Technologies, Santa Clara, CA, USA), respectively. *De novo* sequencing with 2 × 250 bp and 2 × 300 bp reads configuration was carried out on the Illumina MiSeq platform (Illumina, Inc., San Diego, CA, USA) at the Luxembourg Institute of Science and Technology. Complete genomes were assembled from raw sequence data with SPAdes v.3.6.2 (Bankevich et al., 2012) using the “careful” option, and the quality of the assemblies was subsequently assessed with QUAST v2.3 (Gurevich et al., 2013).

To infer the evolutionary relationships between moonmilk strains and their closest relatives, as well as between cave isolates themselves, 16S rRNA-based phylogeny was combined with multilocus sequence analysis (MLSA). For this purpose, along with the 16S rRNA gene, five additional housekeeping genes were selected, namely *atpD*, *gyrB*, *recA*, *rpoB*, and *trpB* (Han et al., 2012). In order to identify these genes within moonmilk genomes, the corresponding nucleotide sequences (16S rRNA) and protein translations (*atpD*, *gyrB*, *recA*, *rpoB*, and *trpB*) were retrieved from the NCBI web portal for three reference strains: *Streptomyces peucetius* AS 4.1799, *Streptomyces pristinaespiralis* ATCC 25486 and *Streptomyces venezuelae* ATCC 10712 (**Supplementary Table S1**). Core alignments were built using MAFFT v7.273 (Katoh and Standley, 2013) with default parameters, then enriched in the corresponding sequences from moonmilk genomes using the software “42” (D. Baurain, to be published elsewhere), which mines genomic contigs for orthologous genes and aligns the (translated) identified sequences on their closest relatives. Enriched alignments were then refined by hand using the ED program of the MUST software package (Philippe, 1993). Finally, protein sequences were turned into nucleotide sequences using the software “1331” (D. Baurain, to be published elsewhere), which uses a protein alignment as a guide to generate the corresponding nucleotide alignment from genomic contigs. The sequences of the five protein coding housekeeping genes for all the moonmilk isolates were deposited in GenBank and the corresponding accession numbers are given in **Supplementary Table S2**, while **Table 1** and **Supplementary Table S3** list the NCBI accession numbers of the 16S rRNA gene sequences.

**Table 1:** The closest relatives, phylogenetic affiliations, phylotype clustering, and isolation origin of the 31 representative moonmilk isolates.

Isolate	Closest relatives	16S rRNA identity % (gaps)	Accession number	Origin of the closest relatives	COL	Md	Phylotype 16S → MLSA
MM1	*S.* sp. CFMR 7 strain CFMR-7*/S. fulvissimus* DSM 40593	99.1 (4)/99.1 (6)	KU714864	Plant (rubber)/unknown	COL3	SN	I ** =** I
MM3	Un. bacterium clone Md-54/Un. bacterium clone 10–355	99.8 (0)/99.8 (0)	KU714892	Soil/soil	COL3	SN	II ** =** II
MM5	*S. scabiei* BCCO 10_524*/S. europaeiscabiei* 08-46-04-2 (#50)	99.7 (0)/99.4 (2)	KU714904	Both plant (potato)	COL3	SN	III ** =** III
MM6	*S.* sp. Mg1*/S.* sp. SXY10	99.7 (0)/99.8 (0)	KU714910	Glacier soil (Alaska)/soil	COL3	SN	IV ** =** IV
MM7	*S.* sp. NEAU-spg16*/S.* sp. A42	99.6 (0)/99.9 (0)	KU714915	Soil/soil	COL3	SN	V = V
MM10	*S.* sp. NEAU-QHHV11*/S.* sp. (Acc.Nr.D63866)	99.1 (4)/98.6 (7)	KU714865	Soil/soil	COL3	SN	VI = VI
MM12	*S. sanglieri* A14*/S.* sp. ME03-5656.2c	99.8 (0)/99.6 (0)	KU714878	Soil/plant (potato)	COL3	SN	VII = VII
MM13	*S. turgidiscabies* ATCC 700248*/S. turgidiscabies* WI04-05A	98.8 (4)/98.7 (4)	KU714882	Both plant (potato)	COL3	SN	VIII = VIII
MM14	*S. anulatus* strain 173826*/S. anulatus* strain 173541	100 (0)/100 (0)	KU714883	Both unknown	COL3	SN	IX = IX
MM17	*S.* sp. Mg1*/S.* sp. SXY10	99.7 (0)/99.8 (0)	KU714885	Glacier soil (Alaska)/soil	COL3	SN	IV → XXVI
MM19	*S.* sp. NEAU-spg16*/S.* sp. A42	99.6 (0)/99.9 (0)	KU714887	Soil/soil	COL3	SN	V → XXVII
MM21	Un. bacterium clone Md-54/Un. bacterium clone 10–355	99.7 (0)/99.7 (0)	KU714888	Soil/soil	COL3	SN	XI = XI
MM23	Un. bacterium clone Md-54/Un. bacterium clone 10–355	99.8 (0)/99.8 (0)	KU714890	Soil/soil	COL3	SN	II → XXVIII
MM24	*S.* sp. ME02-6979.3a*/S.* sp. 1C-HV8	98.3 (5)/98.4 (4)	KU714891	Plant (potato)/animals (ants)	COL3	SN	XII = XII
MM44	Un. bacterium clone Md-54/Un. bacterium clone 10–355	99.7 (0)/99.7 (0)	KU714900	Soil/soil	COL3	SN	XI → XXIX
MM48	*S.* sp. HBUM171258*/S.* sp. Mg1	99.9 (1)/99.6 (0)	KU714903	Unknown/glacier soil (Alaska)	COL3	MMch	XIII = XIII
MM59	*S.* sp. ID05-8D*/S.* sp. ID01-6.2a	99.5 (1)/99.4 (1)	KU714909	Both plant (potato)	COL3	MMch	III → XXX
MM68	*S. turgidiscabies* ATCC 700248*/S. turgidiscabies* WI04-05A	99.0 (2)/99.0 (2)	KU714913	Both plant (potato)	COL3	B-4	XIV = XIV
MM90	*S.* sp. AA58*/S.* sp. AS40	99.5 (4)/99.4 (5)	KU714925	Soil/soil	COL1	ISP4	XV → –
MM99	*S. fulvissimus* DSM 40593*/S.* sp. ME02-6987.2c	99.7 (2)/99.7 (2)	KU714928	Unknown/plant (potato)	COL1	ISP6	XVI = XVI
MM100	*S. sanglieri* A14*/S.* sp. ME03-5656.2c	99.9 (0)/99.5 (0)	KU714866	Soil/plant (potato)	COL1	B-4	XVII = XVII
MM104	*S. scopuliridis* strain SCSIO ZJ46*/S.* sp. AK02-1a	99.2 (0)/99.0 (0)	KU714869	Deep sea/plant (potato)	COL3	ISP6	XVIII = XVIII
MM105	*S. finlayi* strain CB00817*/S. olivoviridis* strain S3	99.4 (6)/99.3 (5)	KU714870	Soil/animals (earthworm)	COL3	ISP6	XIX = XIX
MM106	*S. rishiriensis* strain 1706*/S. fimbriatus* strain cfcc3155	99.0 (0)/98.8 (1)	KU714871	Soil/unknown	COL3	ISP1	XX = XX
MM107	*S. pristinaespiralis* strain HCCB 10218*/S.* sp. NEAU-bt10	98.8 (2)/98.8 (0)	KU714872	Soil/soil	COL3	ISP1	XXI = XXI
MM108	*S.* sp. SXY66*/S.* sp. 1H-TWYE2	100 (0)/99.3 (2)	KU714873	Soil/animals (ants)	COL3	ISP7	XXII = XXII
MM109	*S. lunaelactis* MM109^T^*/S. lunaelactis* MM15	100 (0)/99.9 (0)	KM207217.2	Cave/cave	COL3	ISP7	X = X
MM111	*S.* sp. 1H-TWYE2*/S.* sp. SXY66	99.7 (0)/99.5 (2)	KU714875	Animals (ants)/soil	COL4	ISP6	XXIII = XXIII
MM117	*S.* sp. PAMC26508*/S. pratensis* ATCC 33331	99.7 (0)/99.7 (0)	KU714876	Antarctic lichen/soil	COL4	ISP7	XXIV = XXIV
MM122	*S.* sp. PAMC26508*/S. pratensis* ATCC 33331	100 (0)/100 (0)	KU714879	Antarctic lichen/soil	COL4	B-4	IX → XXXI
MM128	*S.* sp. ZLN234*/S.* sp. SXY66	99.9 (0)/99.0 (4)	KU714881	Glacier soil (Arctic)/soil	COL4	SN	XXV = XXV


*B-4, B-4 agar medium; MMch, minimal medium with 1% chitin; ISP, International Streptomyces Project medium; SN, starch nitrate medium; COL, moonmilk collection site; Md, isolation medium; Un., uncultured. Symbols: -, isolates not included in the MLSA; T, Type strain (Maciejewska et al., 2015).*

In order to profile the potential of moonmilk isolates to biosynthesize secondary metabolites, the genes coding for type I, type II, and type III polyketide synthases (PKS-I, PKS-II, and PKS-III) and non-ribosomal peptide synthetases (NRPS) were recovered from their genomes using antiSMASH v3.0.4 (Weber et al., 2015). Due to the fragmented nature of the genome assemblies (ranging from 318 contigs longer than 1 kb for MM23 to 1416 contigs for MM59) and to the large size of the modular NRPS and PKS-I genes (over 40 kb, Wang et al., 2014), the probability of finding complete coding sequences decreases together with the contig length. Therefore, counting the number of genes or clusters split across several shorter contigs would result in an overestimation of the total amount of such genes. To palliate the absence of fully assembled chromosomes while still collecting meaningful statistics, we decided to apply a cut-off on the length of the contigs selected for analysis (minimum length of 10 kb) and to consider NRPS and PKS-I gene sequences only when they displayed adenylation and acyltransferase domains, respectively. These domains, which are the highly selective gatekeeper enzymes for the incoming monomeric building blocks, are required for the initiation and elongation modules of the NRPS/PKS-I clusters. The number of predicted genes of each category for each individual phylotype is compared to their respective mean antimicrobial activities against Gram-positive, Gram-negative bacteria, and fungi. The accession numbers of NRPS and PKS-I/II/III genes are listed in **Supplementary Table S4**.

### Phylogenetic Analysis

To carry out phylogenetic analysis, in each nucleotide alignment of the six selected housekeeping genes (see above), the sequences from the three reference strains used to mine the moonmilk genomes were removed. Then, positions with missing character states in >5 moonmilk isolates were removed. Finally, the trimmed alignments were concatenated into a single (MLSA) supermatrix of 10,632 nucleotides for 70 isolates using SCaFoS v1.30k (Roure et al., 2007). For 16S rRNA phylogenies, the closest relatives to the moonmilk isolates were recovered by BLAST searches using full-length 16S rRNA sequences, along with the *Streptomyces* isolates from a moonmilk deposit in Siberia (Axenov-Gribanov et al., 2016). For the eight isolates for which the genomes were not sequenced (MM9, MM32, MM39, MM55, MM73, MM88, MM90, MM93), nearly full-length 16S rRNA sequences were obtained using PCR primers and conditions as previously reported (Maciejewska et al., 2015). The 78-moonmilk strain alignment of the 16S rRNA was further processed using the software “two-scalp” (D. Baurain, to be published elsewhere) to integrate 35 additional sequences, corresponding to the 27 (non-redundant) best BLAST hits, 7 Siberian moonmilk sequences, and the sequence of *Saccharopolyspora erythraea*, used as the outgroup.

Both the MLSA supermatrix and the 16S rRNA alignment were submitted to phylogenetic inference using the rapid bootstrap analysis of RAxML v8.1.17 (Stamatakis, 2014; 100 pseudoreplicates) under the model GTR+I+Γ_4_. The resulting MLSA and 16S rRNA trees were first formatted in FigTree v1.4.2^1^ then further arranged using Inkscape v0.91^2^. Patristic distances between moonmilk isolates were derived from the MLSA tree using the TREEPLOT program of the MUST software package (Philippe, 1993).

### Antimicrobial Activity Screening

Antimicrobial activities of one representative of each phylotype deduced from the MLSA, together with MM90 (representing 16S-phylotype XV) were tested using the cross-streak method on two (antifungal test) to five (antibacterial test) different culture media: Mueller Hinton Agar (MHA) (Difco, BD, Franklin Lakes, NJ, USA), Tryptic Soy Agar (TSA) (tryptone 15 g, soybean meal 5 g, NaCl 5 g, agar 15 g; pH 7.3), ISP media No. 7 (Shirling and Gottlieb, 1966), starch nitrate (SN) medium (Waksman and Lechevalier, 1961), and minimal medium (Kieser et al., 2000) supplemented with 25 mM *N*-acetylglucosamine (MM + GlcNAc). Each moonmilk strain was independently inoculated from the mycelium stock as a single line in the center of the square plate and incubated for 7 days at 28°C, before being cross-streaked with bacterial or fungal reference strains.

Antibacterial activities were tested against a range of Gram-positive and Gram-negative bacteria, including *Escherichia coli* (ATCC 25922), *Pseudomonas aeruginosa* (ATCC 27853), *Citrobacter freundii* (ATCC 43864), *Klebsiella pneumoniae* (ATCC 13883), *Bacillus subtilis* (ATCC 19659), *Staphylococcus aureus* (ATCC 25923), and *Micrococcus luteus* (ATCC 9341). Each tested bacteria was cross-streaked perpendicular to the growth of the moonmilk isolate at the distance of 2 cm from one another from a suspension prepared according to EUCAST recommendations (EUCAST, 2015). Briefly, each inoculum was made from the overnight-grown plate of each pathogen in solid LB (Difco, BD, Franklin Lakes, NJ, USA) at 37°C by suspending several morphologically similar colonies in LB broth (Difco, BD, Franklin Lakes, NJ, USA) until the OD_625 nm_ reached 0.08–0.13, corresponding to the McFarland 0.5 standard. The reference bacterial strains were cross-streaked with a cotton swab, the plates incubated overnight at 37°C, and the activities determined by measurement of the inhibition zone (in cm).

Antifungal activities were tested against a range of pathogenic fungi including *Candida albicans* (ATCC 10231), *C. albicans* (azole-resistant routine strain from the National Reference Center for Mycoses, 13-160409-5014), referred as *C*. *albicans* ‘R,’ *Aspergillus fumigatus* (Neqas 1210), *Rasamsonia argillacea* (Neqas 1872), *Penicillium chrysogenum* (Neqas 2068), and *Trichophyton mentagrophytes* (Neqas 1208). Each fungal strain was suspended in water at the density equivalent to 0.5 McFarland, and the obtained fungal suspension was perpendicularly cross-streaked against moonmilk isolates with a distance of 4 cm from one another. The plates were incubated at 37°C, for up to 4 days, and the measurements of inhibition zones (in cm) were recorded every day.

### MM99 Isolate Antifungal Agents Extraction and Purification by High Pressure Liquid Chromatography (HPLC)

*Streptomyces* sp. MM99 was inoculated on 15 Glucose Yeast and Malt medium (GYM; glucose 4 g; yeast extract 4 g; malt extract 10 g; casein enzymatic hydrolysate 1 g; NaCl 2 g; agar 20 g) plates and incubated for 10 days at 28°C. The agar was collected, poured into a flask with an equal volume of ethyl acetate (∼300 ml) and agitated overnight at room temperature for metabolites extraction. Ethyl acetate was collected and pieces of agar were removed by centrifugation (25 min at 4000 rpm) before the solvent was evaporated on a rotary evaporator (IKA RV10 digital, VWR, Radnor, PA, USA). The dried crude extract was resuspended in 4 ml of pure methanol high pressure liquid chromatography (HPLC Barker UHPLC grade). Prior to fractionation by HPLC, the antifungal activity of the total extract was assessed by a disk diffusion assay on a yeast peptone dextrose (YPD; peptone 20 g; yeast extract 10 g; glucose 20 g; agar 15 g) agar plate inoculated with *Saccharomyces cerevisiae* (ATCC 9763; with a cotton swab dipped in a 0.25–0.27 OD_625_ LB suspension). The full extract was then fractionated by HPLC (Waters, Milford, MA, USA) using a Waters 2695 Separations Module (Alliance) with a Waters 2998 Photodiode Array Detector coupled to a Waters Fraction Collector WFC III. The methanol extract was analyzed on a Nucleodur C18ec column (2.0 mm × 150 mm, 5 μm particle size, Macherey-Nagel) at a column temperature of 40°C. Extract separation was achieved by increasing the acetonitrile (Barker, HPLC far UV grade)/water (milliQ filtrated on 0.22 μm) + 0.05% trifluoroacetic acid (TFA, Sequencing grade; Thermo Fisher Scientific, San Jose, CA, USA), ratio (from 0 to 62.5% of acetonitrile during 30 min, then from 62.5 to 100% of acetonitrile during 8 min) at a 300 μl/min flow rate. Online UV absorption measurement was performed from 190 to 800 nm. Data were analyzed using Empower 3 software (Waters, Milford, MA, USA). The obtained extract fractions were subsequently tested for antifungal activities by a disk diffusion assay as described above.

### Compound Identification by Ultra-Performance Liquid Chromatography-Tandem Mass Spectrometry (UPLC-MS/MS)

Fractions displaying antifungal activities revealed by the disk diffusion assay were analyzed by liquid chromatography–tandem mass spectrometry (LC–MS/MS). Briefly, compounds were separated by reverse-phase chromatography using Ultra Performance Liquid chromatography (UPLC IClass, Waters) using a Nucleodur C18ec column (2.0 mm × 150 mm, 5 μm particle size, Macherey-Nagel). Elution was achieved by increasing the acetonitrile/water (milliQ filtrated on 0.22 μm) + 0.05% trifluoroacetic acid ratio (from 0 to 62.5% during 30 min, then from 62.5 to 100% during 8 min) at a 300 μl/min flow rate. On-line UV absorption measurement was performed at 210 and 265 nm and the chromatography system was finally coupled to a Q Exactive Plus hybrid Quadrupole-Orbitrap Mass Spectrometer (Thermo Fisher Scientific, San Jose, CA, USA), operated in positive ion mode and programmed for data-dependent acquisitions. Survey scans were acquired at mass resolving power of 140,000 FWHM (full width at half maximum) from 100 to 1500 m/z (1 × 10^6^ ions accumulation target). The five most intense ions were then selected to perform MS/MS experiments by Higher Energy Collision Dissociation (HCD) fragmentations using stepped normalized collision energy (NCE; 21,2; 25; 28) within 2 amu isolation windows (resolution 17500, 1 × 10^5^ ions accumulation target). A dynamic exclusion was enabled for 10 s. Data were analyzed using Xcalibur v2.2 (Thermo Fisher Scientific, San Jose, CA, USA). Commercial cycloheximide standard (Sigma-Aldrich, St. Louis, MO, USA) was used as a control.

## Results and Discussion

### Isolation of Actinobacteria from Moonmilk Deposits

**FIGURE 2:**
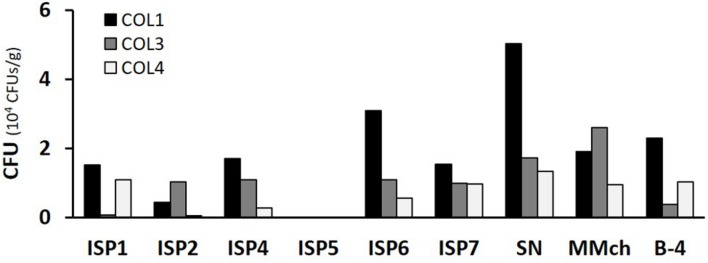
**Total number of CFU obtained from each moonmilk collection point according to the selective media used.** CFU, colony-forming units; ISP, International *Streptomyces* Project; SN, starch nitrate; MMch, minimal medium supplemented with chitin.

Cultivable actinobacterial species were isolated from the three separate moonmilk deposits within Grotte des Collemboles (**Figure 1**). Our cultivation-based screening on Actinobacteria-specific media revealed high population density across the three collection sites studied, with up to 10^4^ CFUs/g (**Figure 2**). No microbial growth could be detected in the ISP5 medium (Glycerol Asparagine Agar), while the highest numbers of CFUs for each sampling point were recorded on the starch nitrate (SN) medium (5 × 10^4^ and 1.3 × 10^4^ CFUs/g in COL1 and COL4, respectively), and on the minimal medium supplemented with chitin (2.6 × 10^4^ CFUs/g in COL3; **Figure 2**). Most of the viable isolates (>50%) were chosen from the starch nitrate medium (**Table 1**; **Supplementary Table S3**), in which the most distinct colony phenotypes were observed. From the 129 colonies initially selected, only 78 were recovered as pure isolates after three rounds of inoculation/cultivation, and were preserved for further studies. This important loss (40%) during the purification steps was expected, as living in an oligotrophic environment necessarily implies nutrient exchange between individuals of the microbiome, a cooperative strategy that was only possible in the non-diluted plates where colonies were originally picked. Inoculation of single colonies thus biased the selection of specimens able to feed only from nutrients present in the synthetic medium, whereas species most adapted to cooperative growth certainly represented a significant part of the isolates lost during the screening procedure.

### Phylogeny of Cultivable Moonmilk-Derived *Streptomyce*s Isolates

When cultivated in liquid cultures, all the isolates formed filamentous pellets, suggesting their affiliation to the genus *Streptomyces* or to other filamentous Actinobacteria (data not shown). BLAST search using either full or nearly full-length 16S rRNA gene sequences indeed revealed that the closest hits to each of the moonmilk-derived isolate were *Streptomyces* species displaying between 98.3 and 100% identity (**Table 1**; **Supplementary Table S3**). Cultivable members of this genus have been previously reported from moonmilk (Cañaveras et al., 1999; Axenov-Gribanov et al., 2016), however isolates belonging to other genera, such as *Amycolatopsis*, *Saccharothrix*, *Acinetobacter*, *Chryseomonas* (Cañaveras et al., 1999), *Arthrobacter* (Rooney et al., 2010), *Pseudonocardia*, *Propionibacterium* (Portillo and Gonzalez, 2011), and *Nocardia* (Axenov-Gribanov et al., 2016), have been found through both culture-dependent and independent approaches. It was therefore unexpected that only *Streptomyces* were isolated across a wide range of Actinobacteria-specific media. One reason for such biased recovery might be that *Streptomyces* are saprophytes accustomed to use a large variety of nutrient sources, and are thus better adapted to grow on the synthetic rich media used in our screening procedure. Secondly, among actinobacterial populations, *Streptomyces* are known for their more rapid growth in comparison to other genera, recognized as rare Actinobacteria, which are isolated much less frequently (Subramani and Aalbersberg, 2013). Therefore, in our *in vitro* culture conditions, *Streptomyces* have probably overtaken nutrients and space over other, slower growing, actinobacterial representatives.

**FIGURE 3:**
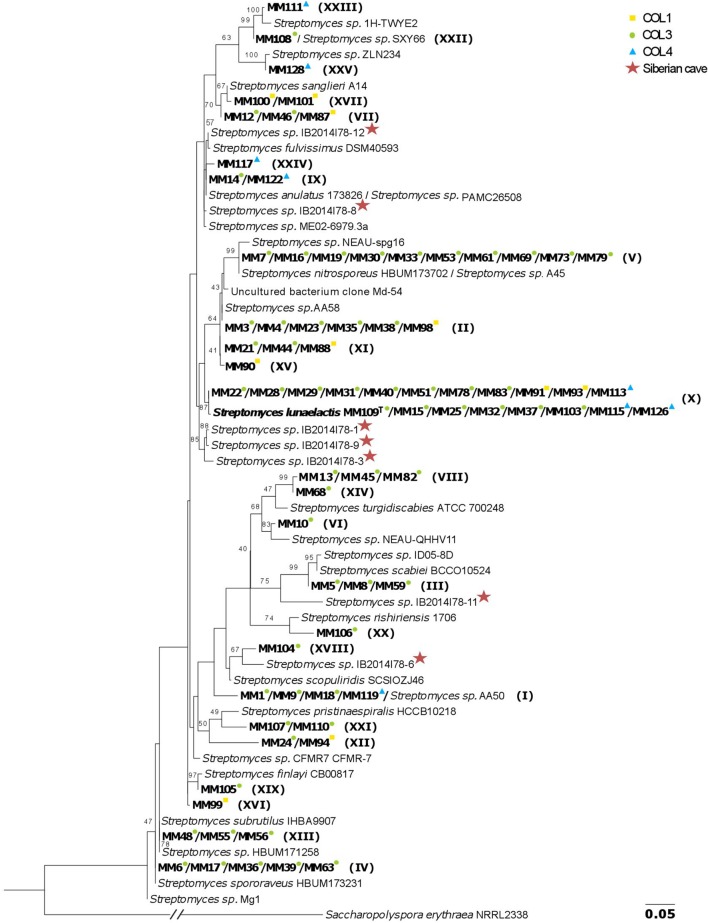
**16S rRNA-based phylogenetic tree of moonmilk cultivable isolates (MM).** The collection points, from which each moonmilk isolate originates are indicated by colored symbols, while the phylotype affiliations are indicated in brackets. The *Streptomyces* isolates originating from moonmilk deposits of the Bolshaya Oreshnaya Cave in Siberia are marked by a star. The alignment of nearly complete 16S rRNA sequences had 1413 unambiguously aligned nucleotide positions for 113 strains. The evolutionary model was GTR+I+Γ_4_ and bootstrap values are based on 100 pseudoreplicates (the bootstrap values below 40% are not displayed). *Saccharopolyspora erythraea* was used as outgroup, and its branch was reduced five times which is indicated by the slanted bars. The scale bar represents 0.05 substitutions per site.

Based on the 16S rRNA tree topology, we grouped the 78 moonmilk isolates into 25 phylogenetic clusters (**Figure 3**; **Table 1**; **Supplementary Table S3**). The largest cluster on the tree (**Figure 3**, phylotype X), delineating the recently described *Streptomyces lunaelactis* species, grouped six novel isolates in addition to the 13 strains reported previously (Maciejewska et al., 2015), which collectively originate from each of the studied sampling site. The clustering of isolates from multiple sampling sites in a single branch was also observed for six other phylotypes, namely phylotypes I, II, VII, IX, XI, and XII, which suggests a widespread occurrence of these isolates within the moonmilk deposits of the cave.

Among the closest environmental *Streptomyces* species deduced from the 16S rRNA BLAST searches, 36 (46%) have been isolated from soils, 12 (15%) from plants, while relatives associated with water ecosystems, lichens, and animals constituted 2 (3%), 2 (3%), and 1 (1%), respectively (**Table 1**; **Supplementary Table S3**). Finally, 19 isolates (24%) were found to be affiliated to the recently described moonmilk species, *S. lunaelactis* (99.9 or 100% 16S rRNA identity), with isolate MM109^T^ selected as the type strain (Maciejewska et al., 2015). The closest known relative of *S. lunaelactis* found through full-length 16S rRNA gene search, *Streptomyces globisporus*, further increases the number of relatives originating from soils to 55 (71%). The high sequence identity of 16S rRNA genes of moonmilk isolates to species from soil, plant, and water environments suggests that the moonmilk may have been seeded with Actinobacterial species brought into the cave from the surface by dripping water (Laiz et al., 1999; Legatzki et al., 2012; Yun et al., 2015). It should be noted that some of our phylotypes are phylogenetically closely related to *Streptomyces* isolates recently identified from moonmilk deposits of the Bolshaya Oreshnaya Cave in Siberia (Axenov-Gribanov et al., 2016). This is particularly the case for phylotypes IX, X, XVIII, and XXIV (**Figure 3**), however the comparison is restricted by the small size of the collection generated from the Siberian cave (limited to seven *Streptomyces* isolates). Nonetheless, this observation supports the idea that we may have identified at least some bacterial species specifically associated with moonmilk speleothems.

**FIGURE 4:**
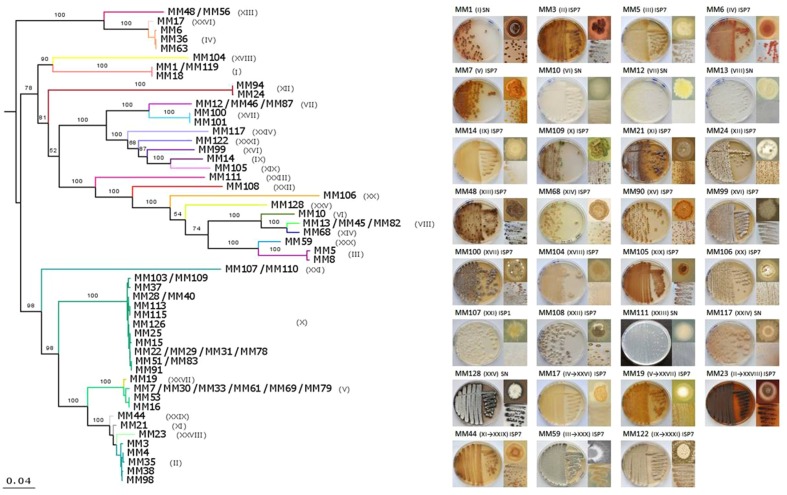
**Multilocus sequence analysis (MLSA) phylogeny of moonmilk isolates and phenotypes of the selected representative of each phylotype.** **(A)** MLSA was based on the full-length sequences of six housekeeping genes (16S rRNA, *atpD*, *recA*, *rpoB*, *gyrB*, *trpB*). Each MLSA-deduced phylotype is indicated by a specific color and the corresponding number in brackets. The supermatrix had 10,632 unambiguously aligned nucleotide positions for 70 isolates. ML phylogenetic inference was carried out as for 16S rRNA alone. The scale bar represents 0.04 substitutions per site. **(B)** Phenotype of the 31 phylotype representative isolates. The MLSA phylotype number is indicated in brackets, with the former, 16S rRNA-deduced phylotypes included. For each isolate, front and back of the Petri dish-grown bacteria are presented together with the phenotype of a single colony.

As 16S rRNA-based phylogeny is known to be insufficient for discriminating between closely related *Streptomyces* species (Guo et al., 2008; Labeda, 2011; Rong and Huang, 2012) and, consequently, could underestimate the true diversity of our collection, a MLSA was additionally performed. Instead of partial gene fragments used previously (Maciejewska et al., 2015), we have retrieved nearly full-length sequences of the selected housekeeping genes (*atpD, rpoB, trpB, gyrB*, and *recA*) from the genomes of 70 out of 78 isolates. As expected, the phylogenetic resolution of the MLSA tree, based on a supermatrix of 10,632 unambiguously aligned nucleotides (including 16S rRNA), was much higher. Hence, some strains previously considered to belong to a single phylotype were separated into several sub-clusters (**Figure 4A**). To clearly delineate the phylotypes, the comparison of the pairwise patristic distances between isolates was taken into account together with the tree topology (**Supplementary Figure S1**; **Figure 4A**). The highest pairwise distance among *S. lunaelactis* strains (0.0096) was considered as the threshold for phylotype assignment since most of these isolates were previously reported to belong to a single species (Maciejewska et al., 2015). Consequently, the number of phylogenetic clusters increased from 25, inferred from the 16S rRNA tree, to 31 based on MLSA. The new phylotypes differentiated through MLSA were represented by MM17 (IV→XXVI), MM19 (V→XXVII), MM23 (II→XXVIII), MM44 (XI→XXIX), MM59 (III→XXX), and MM122 (IX→XXXI) (**Table 1**). To assess whether the distinction of new phylotypes was supported by phenotypic traits, we compared pigment production and colony morphologies between representative isolates of MLSA phylotypes and representative isolates of their former 16S rRNA-based phylotypes. For instance, the separation of isolate MM17 (now phylotype XXVI) from isolates of phylotype IV (represented by MM6) was indeed additionally supported by the phenotype analysis, as unlike MM6 isolate, which produces a dense pink-reddish intracellular pigment, isolate MM17 remains unpigmented when grown on ISP7 (**Figure 4B**). Dissimilar phenotypes were also observed between representative isolates that formerly belonged to the same phylotype, i.e., MM7 and MM19, MM23 and MM3, MM44 and MM21, MM59 and MM5, and MM122 and MM14 (**Figure 4B**).

### Antimicrobial Activity Screening

**FIGURE 5:**
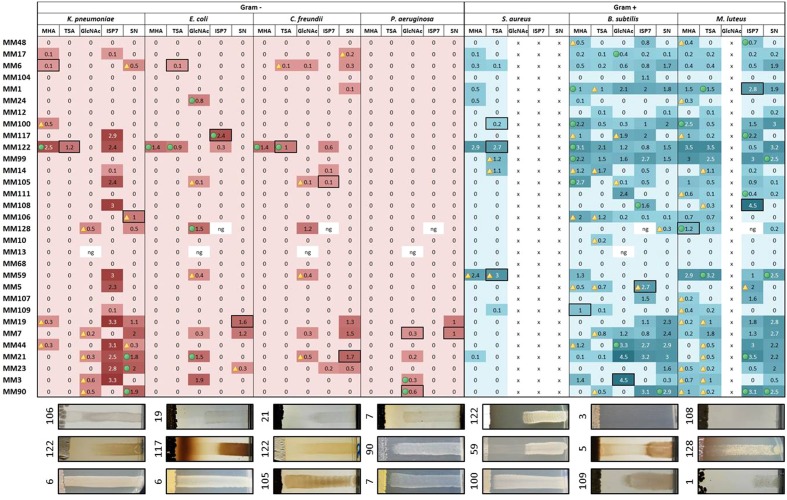
**Heatmap illustrating the strength of the antibacterial activities of the representatives of moonmilk phylotypes.** The size of the inhibition zone (in cm) is indicated for each moonmilk isolate and related to a color scale. Isolates causing impaired growth of the tested bacteria are indicated by a yellow triangle, inhibition effects combining both impaired growth and growth inhibition are marked with a green circle, and clear bactericidal growth inhibition effects are not marked with any symbol. The fields selected with a black border refer to the chosen examples of the cross-streak results displayed below the heatmap. x, the tested pathogen cannot grow in the chosen medium; ng, no or poor growth of the moonmilk isolate.

**FIGURE 6:**
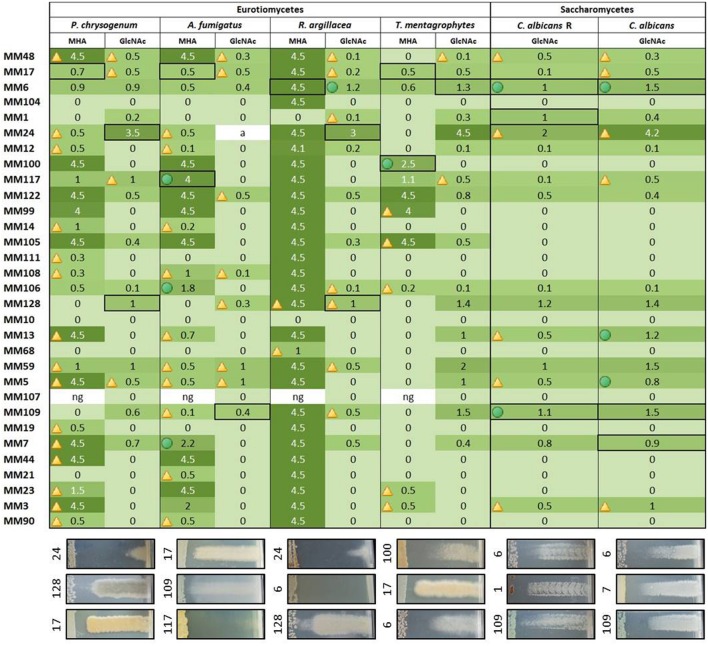
**Heatmap illustrating the strength of the antifungal activities of the representatives of moonmilk phylotypes.** The size of the inhibition zone (in cm) is indicated for each moonmilk isolate and related to a color scale. Isolates causing impaired growth of the tested fungal strains are indicated by a triangle, inhibition effects combining both impaired growth and growth inhibition are marked with a green circle, and clear bactericidal growth inhibition effects are not marked with any symbol. The fields selected with a black border refer to the chosen examples of the cross-streak results displayed below the heatmap. ng, no or poor growth of the moonmilk isolate.

The potential for antimicrobial activity of the moonmilk-derived cultivable isolates was assessed for one representative of each of the 31 phylotypes deduced from the MLSA (see heatmaps in **Figures 5** and **6** for antibacterial and antifungal activities, respectively). Two categories of antimicrobial activities were recorded, i.e., (i) those that fully inhibited the growth (GI, growth inhibition) of the tested reference strains (see **Figure 5** MM122 against *S. aureus* as an example), and (ii) those that allowed only partial growth (IG, impaired growth, see **Figure 5** MM5 against *B. subtilis* as an example).

**FIGURE 7:**
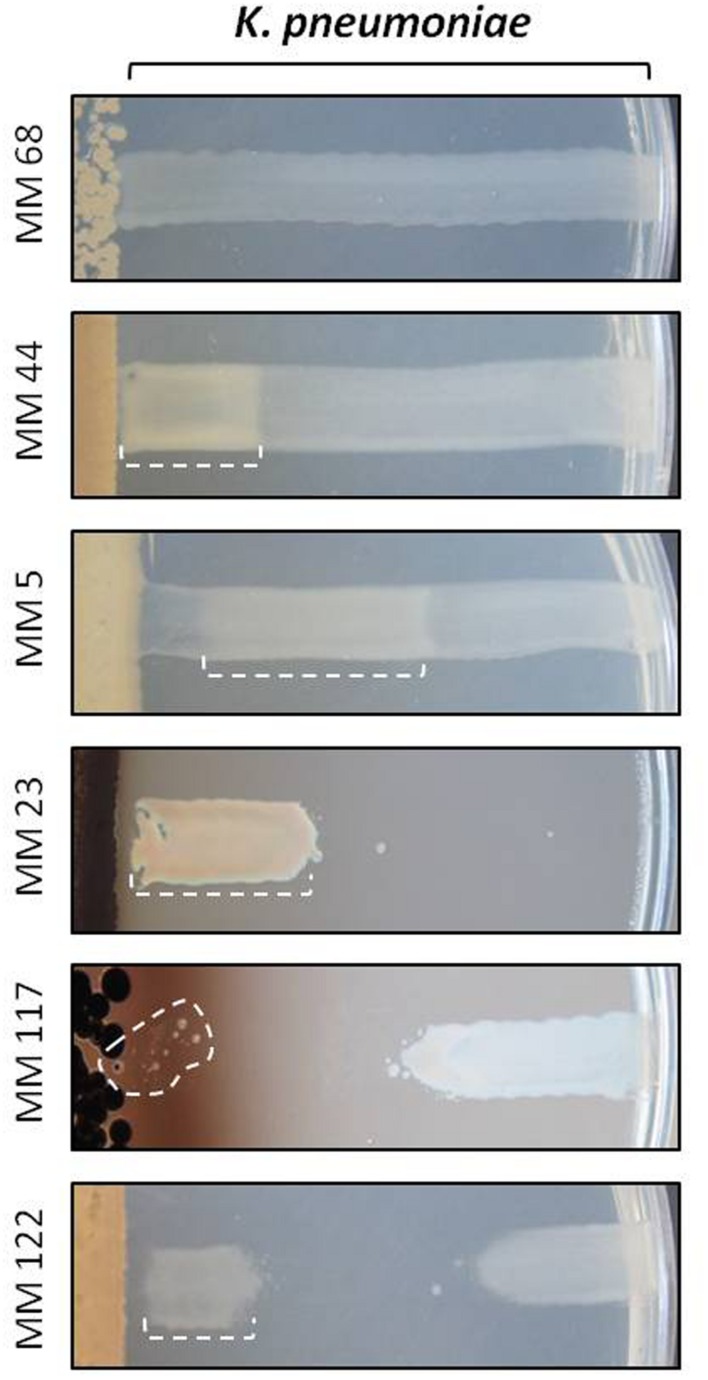
**Illustration of the paradoxical zone phenomenon observed with *Klebsiella pneumoniae*.** Zones with the so-called Eagle or hormesis effects are delineated by a dotted line. Note that for compounds produced by isolate MM117 we only observed the emergence of isolated resistant colonies instead of the wide and confluent zone of growth observed for the other marked cases. MM68 was chosen as a control to show non-affected growth of *K. pneumoniae* on the ISP7 medium.

**FIGURE 8:**
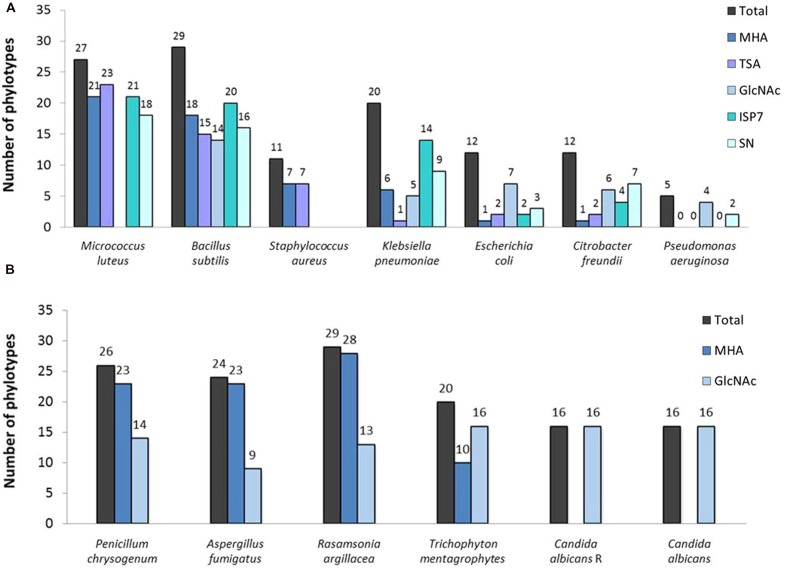
**Total number of the representatives of the phylotypes displaying an activity against **(A)** bacterial and **(B)** fungal strains under each tested condition**.

**FIGURE 9:**
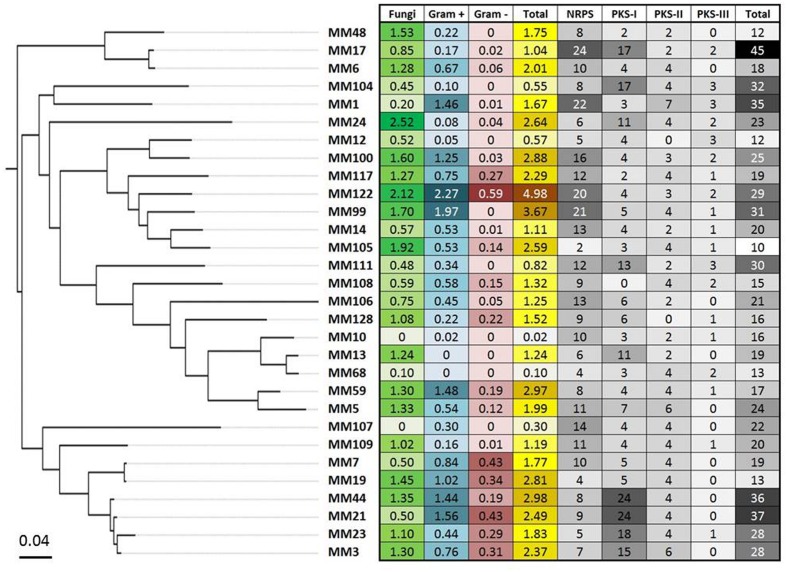
**The global antimicrobial pattern of moonmilk *Streptomyces* isolates and their presumed secondary metabolites biosynthesis potential based on genome mining.** The heatmap plot, correlated with MLSA-based phylogeny, represents the mean of the antifungal and antibacterial (Gram-positive and Gram-negative bacteria) activities for each of the 30 phylotype representatives, together with the sum of those activities. In addition, the greyscale on the heatmap displays the biosynthetic genes content of individual strain, including number of its NRPS, PKS-I, PKS-II, and PKS-III genes, together with their total number.

The screening for antibacterial properties was carried out under five different culture conditions, i.e., the two complex and nutrient rich media (MHA and TSA), the ISP7 and SN media from which most of our strains were isolated (see **Table 1**; **Supplementary Table S3**), and the minimal medium supplemented with GlcNAc, which is a known elicitor of antibiotics under poor culture conditions (Rigali et al., 2008; Światek et al., 2012; Zhu et al., 2014). Results from the antibacterial cross-streak are presented in the heatmap of **Figure 5** (with particular cases highlighted in **Figure 7**), and summarized in **Figures 8A** and **9**. Overall, the moonmilk isolates expectedly displayed a much stronger antibacterial activity (GI and IG) against Gram-positive bacteria (94% of the phylotypes) than against Gram-negative bacteria (71% of the phylotypes). Indeed, 94% of the tested isolates were active against *B. subtilis*, 87% against *M. luteus*, and 36% against *S. aureus*, while 65% were found to show activity against *K. pneumoniae*, 39% against *E. coli*, 39% against *C. freundii* and only 16% against *P. aeruginosa* (**Figures 5** and **8A**). The most active isolate in terms of both strength and spectrum of targeted pathogens was MM122 (**Figures 5** and **9**). Interestingly, a group of four isolates (MM3, MM7, MM21, and MM90) similarly triggered the production of compounds active against Gram-negative bacteria, particularly *P. aeruginosa* and *K. pneumonia*, in the presence of GlcNAc (**Figure 5**). All these isolates branch together in the 16S rRNA and the MLSA-deduced phylogenetic trees (**Figures 3** and **4A**), which suggests that their similar response to the GlcNAc eliciting molecule should be induced by the same signaling pathway and involves similar or identical bioactive compounds. Those isolates together with other closely related moonmilk strains, namely MM19, MM44, and MM23, showed one of the strongest inhibitory profiles against a broad spectrum of pathogens and under most of the tested culture conditions. This suggests that they possibly might share a similar set of antibacterial secondary metabolite clusters. In contrast, MM10, MM13, and MM68, all representatives of closely related phylotypes, did not reveal any antibacterial activity; however, genome mining showed that those three isolates are predicted to possess in total 16, 19, and 13 biosynthetic cluster-associated genes (including NRPS, all types of PKS), respectively (**Figure 9**). This suggests that the absence of antibiotic activity would be rather caused by inappropriate culture conditions for eliciting the production of cryptic antibiotics, rather than a lack of genetic material associated with secondary metabolism.

Another remarkable example evidencing that the antibiotic production highly depends on the culture medium composition is illustrated by the isolate MM108, which did not display any antibacterial activity unless grown on the ISP7 medium, where it induced a strong GI effect against *K. pneumonia*, *B. subtilis*, and *M. luteus* (**Figure 5**).

Strikingly, the ISP7 medium also stimulated unusual growth phenotypes by *K. pneumoniae* to compounds produced by our moonmilk isolates. All different atypical cases observed for this pathogen are presented in **Figure 7**. For instance, beyond the classical GI and the IG effects (see **Figure 5**), we observed that *K. pneumoniae* would grow or be only slightly inhibited near the antimicrobial producing strain, whereas its growth was fully or partially inhibited (for example MM23 in **Figure 7**) or impaired (for example MM5 in **Figure 7**) at a higher distance. This non-linear response to a diffusion gradient of the secreted antibiotics has been described as the Eagle effect (Eagle and Musselman, 1948). According to this phenomenon, the activity of antimicrobial compounds is paradoxically higher at lower concentrations, whereas high concentrations allow growth within the vicinity of *Streptomyces*. The observed growth close to the antimicrobial-producing strain might be due to the onset of the resistance mechanisms, which were reported to be rapidly induced by some antibiotics in *K. pneumoniae* (Zhong et al., 2013; Adams-Sapper et al., 2015). Indeed, several resistant *K. pneumoniae* colonies were detected in the proximity to the moonmilk isolate MM117 (**Figure 7**). Another possible explanation might be that the high production of antimicrobials by certain moonmilk isolates on the ISP7 medium could lead to precipitation and consequently inactivation of the antibacterial agents active against *K. pneumoniae*. Finally, the observed atypical response might be explained by a certain form of the hormesis phenomenon, which states that ‘the dose makes the poison’ (Zhanel et al., 1992; Linares et al., 2006; Allen et al., 2010). Consequently, the produced molecules would effectively suppress the growth of the pathogen, but only at the appropriate concentration ranges (Migliore et al., 2013).

Our collection of cultivable moonmilk *Streptomyces* strains was further assessed for the production of compounds with antifungal activities (**Figure 6**). The corresponding screening was carried out under two different culture conditions, i.e., (i) the rich medium MHA and (ii) the minimal medium supplemented with GlcNAc. The latter medium was again selected as GlcNAc, being the monosaccharide composing chitin, exerts a strong carbon catabolite repression on the induction of the chitinolytic system in *Streptomyces* species (Saito et al., 2007; Colson et al., 2008). This prevents ‘false-positive’ growth inhibition of tested fungi due to enzymatic hydrolysis of their chitin-based cell wall. The results of antifungal screening are presented in **Figure 6** and summarized in **Figures 8B** and **9**. Overall, the antifungal activities were stronger when the *Streptomyces* strains were grown on the rich MHA medium (**Figures 6** and **8B**). Globally, 94% of the tested isolates showed inhibitory activities against fungal strains, with 84% being specifically active against *P. chrysogenum*, 77% against *A. fumigatus*, 94% against *R. argillacea*, 65% against *T*. *mentagrophytes*, and 52% active against both *C. albicans* and its drug-resistant strain, *C*. *albicans* ‘R’ (**Figures 6** and **8B**). The most bioactive isolate in terms of the strength and the number of inhibited fungal strains was found to be MM24, while MM10 and MM107 did not show any suppression under conditions tested (**Figure 6**). The latter two isolates, together with MM68, generally represented the least prolific antimicrobial producers (**Figure 9**). The correlation between phylogenetic inference and detected activities was significant for moonmilk phylotypes MM6 and MM17, as well as MM5 and MM59 (**Figure 6**). Remarkably, most of our isolates displayed very strong growth inhibitory properties against the clinically relevant filamentous fungi – *R. argillacea*. This mold, belonging to the so-called *R. argillacea* species complex, is a causative agent of chronic infections of cystic fibrosis (CF) and chronic granulomatous disease (CGD*)* patients, displaying tolerance to various antifungals (Giraud et al., 2013). That almost all our isolates display high GI activity against *R. argillacea* suggests the involvement of one or more molecules commonly produced by cultivable moonmilk streptomycetes. The identification of active compound(s) is currently under investigation.

**FIGURE 10:**
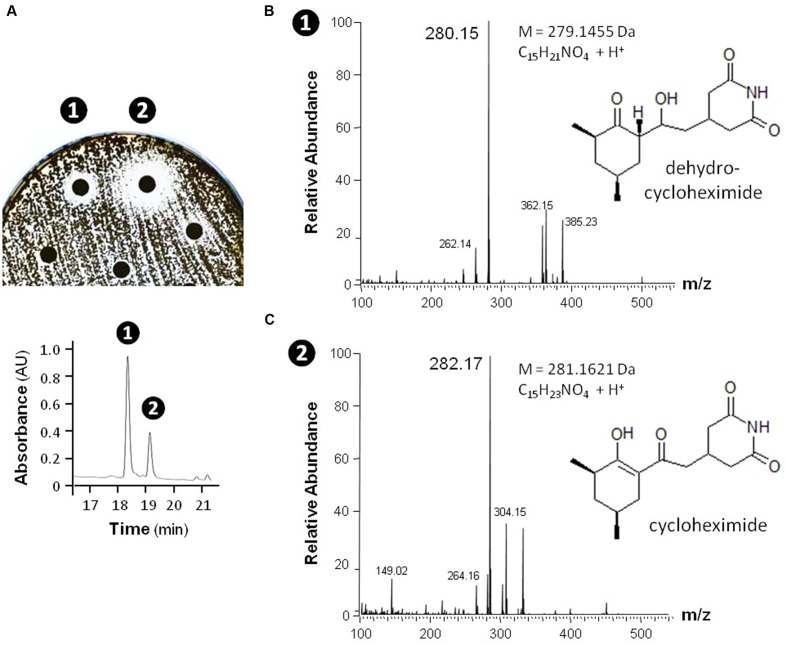
**Identification of cycloheximide and dehydrocylcoheximide as antifungal compounds produced by the isolate MM99.** The antifungal activities of MM99 were detected through disk diffusion assay **(A, top)** of HPLC-separated **(A, bottom)** fractions of the crude metabolites extract. The two bioactive fractions detected were subsequently identified by UPLC-MS/MS to contain mass peaks corresponding to dehydrocycloheximide **(B)** and cycloheximide **(C)**.

In order to provide a first evidence that the observed antimicrobial activities are indeed caused by the production of small inhibitory molecules, and do not result from other factors, such as enzymatic degradation, a single isolate – MM99 – was selected to characterize the compound(s) responsible for its antifungal response. For this purpose, MM99 was cultivated on GYM media, which triggered its antifungal activity, with the media collected for metabolite extraction and separation by HPLC. The two most active HPLC fractions were collected (**Figure 10A**), and subjected to UPLC-ESI-MS/MS analysis, which identified two masses (281.1626 and 279.1470 Da). These two peaks corresponded to cycloheximide and its precursor, dehydrocycloheximide, respectively (**Figures 10B,C**). Cycloheximide is a known inhibitor of eukaryotic protein synthesis, which was first isolated from *Streptomyces griseus* and previously known as actidione (Whiffen et al., 1946; Leach et al., 1947). To additionally confirm that our newly isolated antifungal compound was indeed cycloheximide, we compared the MS/MS fragments profile between the compound isolated from MM99 (**Figure 10C**) and a cycloheximide standard. Identical fragmentation patterns of both samples (**Supplementary Figure S2**), ultimately confirmed that cycloheximide was responsible – at least in part – for the antifungal activity of the isolate MM99. The identification of this single molecule is representative of ongoing efforts that aim to exhaustively identify the bioactive molecules of cultivable *Streptomyces* from moonmilk deposits.

### Genome Mining

**FIGURE 11:**
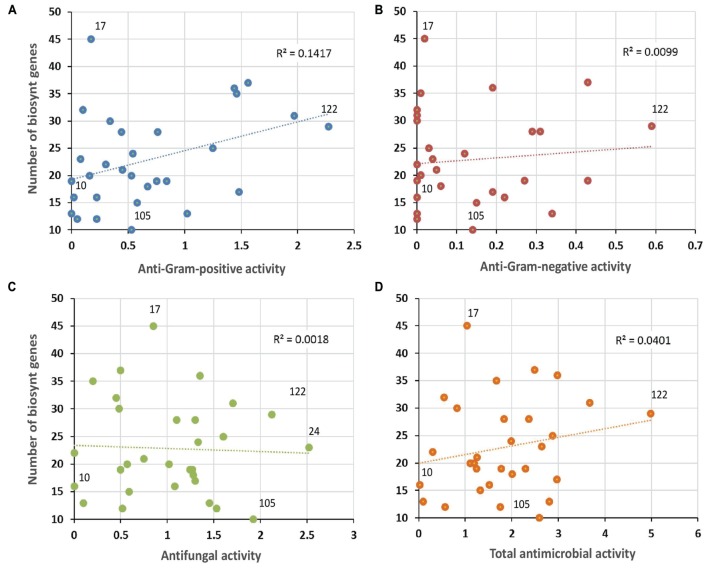
**Correlation between antimicrobial activities and predicted secondary metabolite biosynthetic genes.** The vertical axis refers to the sum of PKS-I, PKS-II, PKS-III, and NRPS biosynthetic genes identified in each phylotype representative by the antiSMASH software. Horizontal axes represent the mean of the antibacterial activity (against Gram-positive bacteria **(A)**, or Gram-negative bacteria **(B)**, the antifungal activity **(C)**, and the total antimicrobial activity **(D)** of each phylotype representative. Note the non-significant R-squared (*R*^2^) values for each plot, which reflect weak correlation between antimicrobial activities and the genetic potential of each isolate to produce secondary metabolites. The isolates that displayed the lowest (MM10) and the highest antibacterial (MM122) and antifungal (MM24) activities, and the smallest (MM105) and the largest (MM17) numbers of secondary metabolite biosynthetic genes, are highlighted in the plots.

Complementary to *in vitro* antimicrobial screenings, we have carried out in parallel a large scale genome mining investigation. For this purpose, the draft genomic assemblies were examined for the presence of biosynthetic genes encoding polyketide synthases – PKSs (including PKS-I, PKS-II, and PKS-III), as well as NRPSs, which are collectively responsible for production of bioactive molecules in Actinobacteria. Notably, 100% of evaluated strains encoded at least two NRPS genes, 97% were found to possess PKS-I, 94% PKS-II, and 48% PKS-III genes (**Figure 9**). This clearly indicates a significant predisposition of moonmilk isolates to secrete bioactive secondary metabolites. The isolate MM17 was found to carry the highest total number of biosynthetic genes (45), while MM105 harbored only 10 of them, being classified as the weakest potential antimicrobial producer (**Figure 9**). Comparison of the biosynthetic genes content and the antimicrobial activities of each individual phylotype did not show significant correlation, with only small relationship observed for anti-Gram positive test (**Figure 11**). Remarkably, MM17 although encoding the highest amount of secondary metabolites genes displayed one of the weakest antimicrobial activities (**Figure 11**). This observation clearly highlights the urgency for finding appropriate culture conditions and triggers which would awaken silent metabolite clusters.

## Conclusion

In this work we have isolated 78 cultivable *Streptomyces* strains from three moonmilk deposits collected in the Grotte des Collemboles, Comblain-au-Pont (Belgium), and assessed their capacity to secrete compounds with antibacterial and antifungal activities. Surprisingly, the isolated strains were found to exclusively belong to the single *Streptomyces* genus, despite other cultivable-dependent and -independent approaches revealed the presence of many other Actinobacteria in moonmilk speleothems (Cañaveras et al., 1999; Rooney et al., 2010; Portillo and Gonzalez, 2011; Axenov-Gribanov et al., 2016). Hence, a more specific protocol for the isolation of rare Actinobacteria is currently tested in order to assess their potential in producing bioactive natural compounds. Phylogenetic analysis revealed the novelty of the selected isolates, and suggests that they might indeed represent indigenous moonmilk populations adapted to life in an inorganic and oligotrophic environment. Overall, antimicrobial screening showed that moonmilk strains were much more active against fungi, than against the bacterial reference strains, which was previously observe for plant-associated actinobacterial isolates (Bascom-Slack et al., 2009; Verma et al., 2009; Zhao et al., 2011). Identification of bioactive compounds is under investigation, particularly molecules active against *R. argillacea.* A complementary, *in silico* genome mining approach additionally revealed a high richness and diversity of secondary metabolite gene clusters, as evidenced by the presence of numerous NRPSs, and PKSs genes. Consequently, an effort has to be made in order to find cues and triggers that would activate the expression of these biosynthetic clusters. Our findings extend the previous data related to broad spectrum antimicrobial activities reported for cave microbiomes of volcanic caves (Cheeptham et al., 2013; Rule and Cheeptham, 2013), and karstic caves (Yücel and Yamaç, 2010), including moonmilk speleothems (Axenov-Gribanov et al., 2016). They provide additional evidence for subterranean systems to be a promising target for bioprospecting for novel bioactive molecules.

## Author Contributions

MM, DA, LM, HB, and MoC collected and processed the samples. MM, DA, LM, AN, MaC, NS, M-PH, MH, DB, and SR performed experiments and analyzed the data. MM, AN, MaC, PD, MH, DB, and SR did bioinformatics, phylogeny analyses, and genome mining. All authors participated to the writing and revision of the manuscript.

## Conflict of Interest Statement

The authors declare that the research was conducted in the absence of any commercial or financial relationships that could be construed as a potential conflict of interest.

## Footnotes

1 http://tree.bio.ed.ac.uk/software/figtree/

2 https://inkscape.org/
