# Supplementary material for: A Phenotypic and Genotypic Analysis of the Antimicrobial Potential of Cultivable Streptomyces Isolated from Cave Moonmilk Deposits
Source: Front Microbiol. 2016 Sep 21;7:1455. doi: 10.3389/fmicb.2016.01455 (PMC5030222; doi:10.3389/fmicb.2016.01455)
Supplement: Supplementary file 1 [file Table_1.DOCX]

**Supplementary Table 1.** Accession numbers of the housekeeping genes of the reference *Streptomyces* strains used to generate core alignments applied for orthologous genes search.

|  | ***trpB*** | ***rpoB*** | ***recA*** | ***gyrB*** | ***atpD*** | **16S rRNA** |
| --- | --- | --- | --- | --- | --- | --- |
| ***S. peucetius* AS 4.1799** | EF055159.1 | EF055104.1 | EF055049.1 | F054994.1 | EF031307.1 | NR_024763.1 |
| ***S. venezuelae* ATCC 10712** | CCA54977 | CCA57631 | CCA58710 | CCA56937 | CCA58311 | NR_102857 |
| ***S. pristinaespiralis* ATCC 25486** | EFH31831 | EDY65539 | EDY65507 | EDY62302 | EDY63418 | - |
