# Supplementary material for: A Phenotypic and Genotypic Analysis of the Antimicrobial Potential of Cultivable Streptomyces Isolated from Cave Moonmilk Deposits
Source: Front Microbiol. 2016 Sep 21;7:1455. doi: 10.3389/fmicb.2016.01455 (PMC5030222; doi:10.3389/fmicb.2016.01455)
Supplement: Supplementary file 2 [file Table_2.DOCX]

**Supplementary Table 2.** Accession numbers of the housekeeping genes of moonmilk isolates.

| **Moonmilk isolate** | **Accession numbers** | | | | |
| --- | --- | --- | --- | --- | --- |
|  | ***trpB*** | ***atpD*** | ***gyrB*** | ***recA*** | ***rpoB*** |
| **MM1** | KX503709 | KX503429 | KX503639 | KX503499 | KX503569 |
| **MM3** | KX503724 | KX503444 | KX503654 | KX503514 | KX503584 |
| **MM4** | KX503730 | KX503450 | KX503660 | KX503520 | KX503590 |
| **MM5** | KX503735 | KX503455 | KX503665 | KX503525 | KX503595 |
| **MM6** | KX503740 | KX503460 | KX503670 | KX503530 | KX503600 |
| **MM7** | KX503743 | KX503463 | KX503673 | KX503533 | KX503603 |
| **MM8** | KX503747 | KX503467 | KX503677 | KX503537 | KX503607 |
| **MM10** | KX503691 | KX503411 | KX503621 | KX503481 | KX503551 |
| **MM12** | KX503701 | KX503421 | KX503631 | KX503491 | KX503561 |
| **MM13** | KX503702 | KX503422 | KX503632 | KX503492 | KX503562 |
| **MM14** | KX503703 | KX503423 | KX503633 | KX503493 | KX503563 |
| **MM15** | KX503704 | KX503424 | KX503634 | KX503494 | KX503564 |
| **MM16** | KX503705 | KX503425 | KX503635 | KX503495 | KX503565 |
| **MM17** | KX503706 | KX503426 | KX503636 | KX503496 | KX503566 |
| **MM18** | KX503707 | KX503427 | KX503637 | KX503497 | KX503567 |
| **MM19** | KX503708 | KX503428 | KX503638 | KX503498 | KX503568 |
| **MM21** | KX503710 | KX503430 | KX503640 | KX503500 | KX503570 |
| **MM22** | KX503711 | KX503431 | KX503641 | KX503501 | KX503571 |
| **MM23** | KX503712 | KX503432 | KX503642 | KX503502 | KX503572 |
| **MM24** | KX503713 | KX503433 | KX503643 | KX503503 | KX503573 |
| **MM25** | KX503714 | KX503434 | KX503644 | KX503504 | KX503574 |
| **MM28** | KX503715 | KX503435 | KX503645 | KX503505 | KX503575 |
| **MM29** | KX503716 | KX503436 | KX503646 | KX503506 | KX503576 |
| **MM30** | KX503717 | KX503437 | KX503647 | KX503507 | KX503577 |
| **MM31** | KX503718 | KX503438 | KX503648 | KX503508 | KX503578 |
| **MM33** | KX503719 | KX503439 | KX503649 | KX503509 | KX503579 |
| **MM35** | KX503720 | KX503440 | KX503650 | KX503510 | KX503580 |
| **MM36** | KX503721 | KX503441 | KX503651 | KX503511 | KX503581 |
| **MM37** | KX503722 | KX503442 | KX503652 | KX503512 | KX503582 |
| **MM38** | KX503723 | KX503443 | KX503653 | KX503513 | KX503583 |
| **MM40** | KX503725 | KX503445 | KX503655 | KX503515 | KX503585 |
| **MM44** | KX503726 | KX503446 | KX503656 | KX503516 | KX503586 |
| **MM45** | KX503727 | KX503447 | KX503657 | KX503517 | KX503587 |
| **MM46** | KX503728 | KX503448 | KX503658 | KX503518 | KX503588 |
| **MM48** | KX503729 | KX503449 | KX503659 | KX503519 | KX503589 |
| **MM51** | KX503731 | KX503451 | KX503661 | KX503521 | KX503591 |
| **MM53** | KX503732 | KX503452 | KX503662 | KX503522 | KX503592 |
| **MM56** | KX503733 | KX503453 | KX503663 | KX503523 | KX503593 |
| **MM59** | KX503734 | KX503454 | KX503664 | KX503524 | KX503594 |
| **MM61** | KX503736 | KX503456 | KX503666 | KX503526 | KX503596 |
| **MM63** | KX503737 | KX503457 | KX503667 | KX503527 | KX503597 |
| **MM68** | KX503738 | KX503458 | KX503668 | KX503528 | KX503598 |
| **MM69** | KX503739 | KX503459 | KX503669 | KX503529 | KX503599 |
| **MM78** | KX503741 | KX503461 | KX503671 | KX503531 | KX503601 |
| **MM79** | KX503742 | KX503462 | KX503672 | KX503532 | KX503602 |
| **MM82** | KX503744 | KX503464 | KX503674 | KX503533 | KX503604 |
| **MM83** | KX503745 | KX503465 | KX503675 | KX503535 | KX503605 |
| **MM87** | KX503746 | KX503466 | KX503676 | KX503536 | KX503606 |
| **MM91** | KX503748 | KX503468 | KX503678 | KX503538 | KX503608 |
| **MM94** | KX503749 | KX503469 | KX503679 | KX503539 | KX503609 |
| **MM98** | KX503750 | KX503470 | KX503680 | KX503540 | KX503610 |
| **MM99** | KX503751 | KX503471 | KX503681 | KX503541 | KX503611 |
| **MM100** | KX503682 | KX503402 | KX503612 | KX503472 | KX503542 |
| **MM101** | KX503683 | KX503403 | KX503613 | KX503473 | KX503543 |
| **MM103** | KX503684 | KX503404 | KX503614 | KX503474 | KX503544 |
| **MM104** | KX503685 | KX503405 | KX503615 | KX503475 | KX503545 |
| **MM105** | KX503686 | KX503406 | KX503616 | KX503476 | KX503546 |
| **MM106** | KX503687 | KX503407 | KX503617 | KX503477 | KX503547 |
| **MM107** | KX503688 | KX503408 | KX503618 | KX503478 | KX503548 |
| **MM108** | KX503689 | KX503409 | KX503619 | KX503479 | KX503549 |
| **MM109** | KX503690 | KX503410 | KX503620 | KX503480 | KX503550 |
| **MM110** | KX503692 | KX503412 | KX503622 | KX503482 | KX503552 |
| **MM111** | KX503693 | KX503413 | KX503623 | KX503483 | KX503553 |
| **MM113** | KX503694 | KX503414 | KX503624 | KX503484 | KX503554 |
| **MM115** | KX503695 | KX503415 | KX503625 | KX503485 | KX503555 |
| **MM117** | KX503696 | KX503416 | KX503626 | KX503486 | KX503556 |
| **MM119** | KX503697 | KX503417 | KX503627 | KX503487 | KX503557 |
| **MM122** | KX503698 | KX503418 | KX503628 | KX503488 | KX503558 |
| **MM126** | KX503699 | KX503419 | KX503629 | KX503489 | KX503559 |
| **MM128** | KX503700 | KX503420 | KX503630 | KX503490 | KX503560 |
