# Supplementary material for: A Phenotypic and Genotypic Analysis of the Antimicrobial Potential of Cultivable Streptomyces Isolated from Cave Moonmilk Deposits
Source: Front Microbiol. 2016 Sep 21;7:1455. doi: 10.3389/fmicb.2016.01455 (PMC5030222; doi:10.3389/fmicb.2016.01455)
Supplement: Supplementary file 3 [file Table_3.DOCX]

| **Supplementary Table 3.** The closest relatives, phylogenetic affiliations, phylotype clustering and source of isolation of the 78 moonmilk isolates. The representatives of MLSA-based phylotypes are highlighted in grey. | | | | | | | |
| --- | --- | --- | --- | --- | --- | --- | --- |
| **Isolate** | **Closest relatives** | **16S rRNA**  **identity % (gaps)** | **Accession number** | **Origin of the closest relatives** | **COL** | **Md** | **Phylotype**  **16S → MLSA** |
| MM1 | *S. sp.* CFMR 7 strain CFMR-7 */ S. fulvissimus* DSM 40593 | 99.1 (4) / 99.1 (6) | KU714864 | plant (rubber) / unknown | COL3 | SN | I **=** I |
| MM3 | Un. bacterium clone Md-54 / Un. bacterium clone 10-355 | 99.8 (0) / 99.8 (0) | KU714892 | soil / soil | COL3 | SN | II **=** II |
| MM4 | *S. sp.* AA58 */ S. sp.* AS40 | 99.9 (1) / 99.8 (2) | KU714899 | soil / soil | COL3 | SN | II **=** II |
| MM5 | *S. scabiei* BCCO 10_524 */ S. europaeiscabiei* 08-46-04-2 (#50) | 99.7 (0) / 99.4 (2) | KU714904 | both plant (potato) | COL3 | SN | III **=** III |
| MM6 | *S. sp.* Mg1 */ S. sp.* SXY10 | 99.7 (0) / 99.8 (0) | KU714910 | glacier soil (Alaska) / soil | COL3 | SN | IV **=** IV |
| MM7 | *S. sp.* NEAU-spg16 */ S. sp.* A42 | 99.6 (0) / 99.9 (0) | KU714915 | soil / soil | COL3 | SN | V = V |
| MM8 | *S. sp.* ID05-8D */ S. sp.* ID01-6.2a | 99.5 (1) / 99.4 (1) | KU714919 | both plant (potato) | COL3 | SN | III = III |
| MM9 | *S. sp.* AA50 */ S. sp.* AS42 | 99.9 (1) / 99.8 (0) | KU714924 | soil / soil | COL3 | SN | I **→** - |
| MM10 | *S. sp.* NEAU-QHHV11 */ S. sp.* (Acc.Nr.D63866) | 99.1 (4) / 98.6 (7) | KU714865 | soil / soil | COL3 | SN | VI = VI |
| MM12 | *S. sanglieri* A14 */ S. sp.* ME03-5656.2c | 99.8 (0) / 99.6 (0) | KU714878 | soil / plant (potato) | COL3 | SN | VII = VII |
| MM13 | *S. turgidiscabies* ATCC 700248 */ S. turgidiscabies* WI04-05A | 98.8 (4) / 98.7 (4) | KU714882 | both plant (potato) | COL3 | SN | VIII = VIII |
| MM14 | *S. anulatus* strain 173826 */ S. anulatus* strain 173541 | 100 (0) / 100 (0) | KU714883 | both unknown | COL3 | SN | IX = IX |
| MM15 | *S. lunaelactis* MM15 */ S. lunaelactis* MM109^T^ | 100 (0) / 99.9 (0) | KJ862783.2 | cave / cave | COL3 | SN | X = X |
| MM16 | *S. sp.* A45 */* Un. bacterium clone A1-257 | 99.8 (3) / 99.7 (3) | KU714884 | soil / soil | COL3 | SN | V = V |
| MM17 | *S. sp.* Mg1 */ S. sp.* SXY10 | 99.7 (0) / 99.8 (0) | KU714885 | glacier soil (Alaska) / soil | COL3 | SN | IV **→** XXVI |
| MM18 | *S. sp.* CFMR 7 strain CFMR-7 */ S. fulvissimus* DSM 40593 | 99.1 (4) / 99.1 (6) | KU714886 | plant (rubber) / unknown | COL3 | SN | I = I |
| MM19 | *S. sp.* NEAU-spg16 */ S. sp.* A42 | 99.6 (0) / 99.9 (0) | KU714887 | soil / soil | COL3 | SN | V **→** XXVII |
| MM21 | Un. bacterium clone Md-54 / Un. bacterium clone 10-355 | 99.7 (0) / 99.7 (0) | KU714888 | soil / soil | COL3 | SN | XI = XI |
| MM22 | *S. lunaelactis* MM15 */ S. lunaelactis* MM109^T^ | 100 (0) / 99.9 (0) | KU714889 | cave / cave | COL3 | SN | X = X |
| MM23 | Un. bacterium clone Md-54 / Un. bacterium clone 10-355 | 99.8 (0) / 99.8 (0) | KU714890 | soil / soil | COL3 | SN | II **→** XXVIII |
| MM24 | *S. sp.* ME02-6979.3a */ S. sp.* 1C-HV8 | 98.3 (5) / 98.4 (4) | KU714891 | plant (potato) / animals (ants) | COL3 | SN | XII = XII |
| MM25 | *S. lunaelactis* MM25 */ S. lunaelactis* MM15 | 100 (0) / 99.9 (0) | KJ862784.2 | cave / cave | COL3 | SN | X = X |
| MM28 | *S. lunaelactis* MM28 */ S. lunaelactis* MM109^T^ | 100 (0) / 99.9 (0) | KJ862785.2 | cave / cave | COL3 | SN | X = X |
| MM29 | *S. lunaelactis* MM29 */ S. lunaelactis* MM109^T^ | 100 (0) / 99.9 (0) | KJ862786.2 | cave / cave | COL3 | SN | X = X |
| MM30 | *S. sp.* NEAU-spg16 */ S. sp.* A42 | 99.6 (0) / 99.9 (0) | KU714893 | soil / soil | COL3 | SN | V = V |
| MM31 | *S. lunaelactis* MM31 */ S. lunaelactis* MM109^T^ | 100 (0) / 99.9 (0) | KJ862787.2 | cave / cave | COL3 | SN | X = X |
| MM32 | *S. lunaelactis* MM32 */ S. lunaelactis* MM15 | 100 (0) / 99.9 (0) | KJ862788.2 | cave / cave | COL3 | SN | X = X |
| MM33 | *S. sp.* NEAU-spg16 */ S. sp.* A42 | 99.6 (0) / 99.9 (0) | KU714894 | soil / soil | COL3 | SN | V = V |
| MM35 | Un. bacterium clone Md-54 / Un. bacterium clone 10-355 | 99.8 (0) / 99.8 (0) | KU714895 | soil / soil | COL3 | SN | II = II |
| MM36 | *S. sp.* Mg1 */ S. sp.* SXY10 | 99.7 (0) / 99.8 (0) | KU714896 | glacier soil (Alaska) / soil | COL3 | SN | IV = IV |
| MM37 | *S. lunaelactis* MM37 */ S. lunaelactis* MM15 | 100 (0) / 99.9 (0) | KJ862789.2 | cave / cave | COL3 | SN | X = X |
| MM38 | Un. bacterium clone Md-54 / Un. bacterium clone 10-355 | 99.8 (0) / 99.8 (0) | KU714897 | soil / soil | COL3 | SN | II = II |
| MM39 | *S. spororaveus* HBUM173231 */ S. spororaveus* 173620 | 100 (0) / 100 (0) | KU714898 | both unknown | COL3 | SN | IV **→** - |
| MM40 | *S. lunaelactis* MM40 */ S. lunaelactis* MM109^T^ | 100 (0) / 99.9 (0) | KJ862790.2 | cave / cave | COL3 | SN | X = X |
| MM44 | Un. bacterium clone Md-54 / Un. bacterium clone 10-355 | 99.7 (0) / 99.7 (0) | KU714900 | soil / soil | COL3 | SN | XI **→** XXIX |
| MM45 | *S. turgidiscabies* ATCC 700248 */ S. turgidiscabies* WI04-05A | 98.8 (4) / 98.7 (4) | KU714901 | both plant (potato) | COL3 | SN | VIII = VIII |
| MM46 | *S. sanglieri* A14 */ S. sp.* ME03-5656.2c | 99.8 (0) / 99.6 (0) | KU714902 | soil / plant (potato) | COL3 | SN | VII = VII |
| MM48 | *S. sp.* HBUM171258 */ S. sp.* Mg1 | 99.9 (1) / 99.6 (0) | KU714903 | unknown / glacier soil (Alaska) | COL3 | MMch | XIII = XIII |
| MM51 | *S. lunaelactis* MM15 */ S. lunaelactis* MM109^T^ | 100 (0) / 99.9 (0) | KU714905 | cave / cave | COL3 | MMch | X = X |
| MM53 | *S. sp.* NEAU-spg16 */ S. sp.* A42 | 99.6 (0) / 99.9 (0) | KU714906 | soil / soil | COL3 | MMch | V = V |
| MM55 | *S. subrutilus* strain IHBA 9907 */ S. cirratus* strain IHBA 9837 | 100 (0) / 100 (0) | KU714907 | both lake (Himalayas) | COL3 | MMch | XIII **→** - |
| MM56 | *S. sp.* HBUM171258 */ S. sp.* Mg1 | 99.9 (1) / 99.6 (0) | KU714908 | unknown / glacier soil (Alaska) | COL3 | MMch | XIII = XIII |
| MM59 | *S. sp.* ID05-8D */ S. sp.* ID01-6.2a | 99.5 (1) / 99.4 (1) | KU714909 | both plant (potato) | COL3 | MMch | III **→** XXX |
| MM61 | *S. sp.* NEAU-spg16 */ S. sp.* A42 | 99.6 (0) / 99.9 (0) | KU714911 | soil / soil | COL3 | MMch | V = V |
| MM63 | *S. sp.* Mg1 */ S. sp.* SXY10 | 99.7 (0) / 99.8 (0) | KU714912 | glacier soil (Alaska) / soil | COL3 | MMch | IV = IV |
| MM68 | *S. turgidiscabies* ATCC 700248 */ S. turgidiscabies* WI04-05A | 99.0 (2) / 99.0 (2) | KU714913 | both plant (potato) | COL3 | B-4 | XIV = XIV |
| MM69 | *S. sp.* NEAU-spg16 */ S. sp.* A42 | 99.6 (0) / 99.9 (0) | KU714914 | soil / soil | COL3 | B-4 | V = V |
| MM73 | *S. nitrosporeus* HBUM173702 */ S. nitrosporeus* 173836 | 100 (0) / 100 (0) | KU714916 | both unknown | COL3 | B-4 | V **→** - |
| MM78 | *S. lunaelactis* MM15 */ S. lunaelactis* MM109^T^ | 100 (0) / 99.9 (0) | KU714917 | cave / cave | COL3 | ISP2 | X = X |
| MM79 | *S. sp.* NEAU-spg16 */ S. sp.* A42 | 99.6 (0) / 99.9 (0) | KU714918 | soil / soil | COL3 | ISP2 | V = V |
| MM82 | *S. turgidiscabies* ATCC 700248 */ S. turgidiscabies* WI04-05A | 98.8 (4) / 98.7 (4) | KU714920 | both plant (potato) | COL3 | ISP2 | VIII = VIII |
| MM83 | *S. lunaelactis* MM15 */ S. lunaelactis* MM109^T^ | 100 (0) / 99.9 (0) | KU714921 | cave / cave | COL3 | ISP2 | X = X |
| MM87 | *S. sanglieri* A14 */ S. sp.* ME03-5656.2c | 99.8 (0) / 99.6 (0) | KU714922 | soil / plant (potato) | COL1 | ISP4 | VII = VII |
| MM88 | *S. sp.* AA58 */ S. sp.* AS40 | 99.8 (2) / 99.6 (4) | KU714923 | soil / soil | COL1 | ISP4 | XI **→** - |
| MM90 | *S. sp.* AA58 */ S. sp.* AS40 | 99.5 (4) / 99.4 (5) | KU714925 | soil / soil | COL1 | ISP4 | XV **→** - |
| MM91 | *S. lunaelactis* MM91 */ S. lunaelactis* MM109^T^ | 100 (0) / 99.9 (0) | KJ862791.2 | cave / cave | COL1 | ISP7 | X = X |
| MM93 | *S. lunaelactis* MM93 */ S. lunaelactis* MM109^T^ | 100 (0) / 99.9 (0) | KJ862792.2 | cave / cave | COL1 | ISP7 | X = X |
| MM94 | *S. sp.* ME02-6979.3a */ S. sp.* 1C-HV8 | 98.3 (5) / 98.4 (4) | KU714926 | plant (potato) / animals (ants) | COL1 | ISP7 | XII = XII |
| MM98 | Un. bacterium clone Md-54 */* Un. bacterium clone 10-355 | 99.8 (0) / 99.8 (0) | KU714927 | soil / soil | COL1 | ISP6 | II = II |
| MM99 | *S. fulvissimus* DSM 40593 */ S. sp.* ME02-6987.2c | 99.7 (2) / 99.7 (2) | KU714928 | unknown / plant (potato) | COL1 | ISP6 | XVI = XVI |
| MM100 | *S. sanglieri* A14 */ S. sp.* ME03-5656.2c | 99.9 (0) / 99.5 (0) | KU714866 | soil / plant (potato) | COL1 | B-4 | XVII = XVII |
| MM101 | *S. sanglieri* A14 */ S. sp.* ME03-5656.2c | 99.9 (0) / 99.5 (0) | KU714867 | soil / plant (potato) | COL1 | B-4 | XVII = XVII |
| MM103 | *S.lunaelactis* MM109^T^ */ S.lunaelactis* MM15 | 100 (0) / 99.9 (0) | KU714868 | cave / cave | COL3 | SN | X = X |
| MM104 | *S. scopuliridis* strain SCSIO ZJ46 */ S. sp.* AK02-1a | 99.2 (0) / 99.0 (0) | KU714869 | deep sea / plant (potato) | COL3 | ISP6 | XVIII = XVIII |
| MM105 | *S. finlayi* strain CB00817 */ S. olivoviridis* strain S3 | 99.4 (6) / 99.3 (5) | KU714870 | soil / animals (earthworm) | COL3 | ISP6 | XIX = XIX |
| MM106 | *S. rishiriensis* strain 1706 */ S. fimbriatus* strain cfcc3155 | 99.0 (0) / 98.8 (1) | KU714871 | soil / unknown | COL3 | ISP1 | XX = XX |
| MM107 | *S. pristinaespiralis* strain HCCB 10218 */ S. sp.* NEAU-bt10 | 98.8 (2) / 98.8 (0) | KU714872 | soil / soil | COL3 | ISP1 | XXI = XXI |
| MM108 | *S. sp.* SXY66 */ S. sp.* 1H-TWYE2 | 100 (0) / 99.3 (2) | KU714873 | soil / animals (ants) | COL3 | ISP7 | XXII = XXII |
| MM109 | *S. lunaelactis* MM109^T^ */ S. lunaelactis* MM15 | 100 (0) / 99.9 (0) | KM207217.2 | cave / cave | COL3 | ISP7 | X = X |
| MM110 | *S. pristinaespiralis* strain HCCB 10218 */ S. sp.* NEAU-bt10 | 98.8 (2) / 98.8 (0) | KU714874 | soil / soil | COL3 | ISP1 | XXI = XXI |
| MM111 | *S. sp.* 1H-TWYE2 */ S. sp.* SXY66 | 99.7 (0) / 99.5 (2) | KU714875 | animals (ants) / soil | COL4 | ISP6 | XXIII = XXIII |
| MM113 | *S. lunaelactis* MM113 */ S. lunaelactis* MM15 | 100 (0) / 99.9 (0) | KJ862794.2 | cave / cave | COL4 | ISP7 | X = X |
| MM115 | *S. lunaelactis* MM115 */ S. lunaelactis* MM15 | 100 (0) / 99.9 (0) | KJ862795.2 | cave / cave | COL4 | ISP7 | X = X |
| MM117 | *S. sp.* PAMC26508 */ S. pratensis* ATCC 33331 | 99.7 (0) / 99.7 (0) | KU714876 | Antarctic lichen / soil | COL4 | ISP7 | XXIV = XXIV |
| MM119 | *S. sp.* CFMR 7 strain CFMR-7 */ S. fulvissimus* DSM 40593 | 99.1 (4) / 99.1 (6) | KU714877 | plant (rubber) / unknown | COL4 | ISP7 | I = I |
| MM122 | *S. sp.* PAMC26508 */ S. pratensis* ATCC 33331 | 100 (0) / 100 (0) | KU714879 | Antarctic lichen / soil | COL4 | B-4 | IX **→** XXXI |
| MM126 | *S. lunaelactis* MM109^T^ */ S. lunaelactis* MM15 | 100 (0) / 99.9 (0) | KU714880 | cave / cave | COL4 | SN | X = X |
| MM128 | *S. sp.* ZLN234 */ S. sp.* SXY66 | 99.9 (0) / 99.0 (4) | KU714881 | glacier soil (Arctic) / soil | COL4 | SN | XXV = XXV |

The representative isolate of each MLSA-deduced phylotype is highlighted in grey. Abbreviations: B-4, B-4 agar medium; MMch, minimal medium with 1% chitin; COL, moonmilk collection site; ISP, International *Streptomyces* Project medium; Md, isolation medium; SN, starch nitrate medium; Un., uncultured. Symbols: -, isolates not included in the MLSA; ^T^, Type strain (Maciejewska et al., 2015).
