# Supplementary material for: A Phenotypic and Genotypic Analysis of the Antimicrobial Potential of Cultivable Streptomyces Isolated from Cave Moonmilk Deposits
Source: Front Microbiol. 2016 Sep 21;7:1455. doi: 10.3389/fmicb.2016.01455 (PMC5030222; doi:10.3389/fmicb.2016.01455)
Supplement: Supplementary file 4 [file Table_4.DOCX]

**Supplementary Table 4.** Accession numbers of the genes coding for type I, type II, and type III polyketide synthases (PKS-I, PKS-II, and PKS-III) and non-ribosomal peptide synthetases (NRPS) of moonmilk isolates.

| **Moonmilk isolate** | **Accession numbers** | | | |
| --- | --- | --- | --- | --- |
|  | ***NRPS*** | ***PKS-I*** | ***PKS-II*** | ***PKS-III*** |
| **MM48** | KX707963,KX707964  KX707965  KX707966  KX707967  KX707968  KX707969  KX707970  KX707971 | KX707972  KX707973 | KX707974  KX707975 |  |
| **MM17** | KX708118  KX708119  KX708120  KX708121  KX708122  KX708123  KX708124  KX708125  KX708126  KX708127  KX708128  KX708129  KX708130  KX708131  KX708132  KX708133  KX708134  KX708135  KX708136  KX708137  KX708138  KX708139  KX708140  KX708141 | KX708142  KX708143  KX708144  KX708145  KX708146  KX708147  KX708148  KX708149  KX708150  KX708151  KX708152  KX708153  KX708154  KX708155  KX708156  KX708157  KX708158 | KX708159  KX708160 | KX708161  KX708162 |
| **MM6** | KX707790  KX707791  KX707792  KX707793  KX707794  KX707795  KX707796  KX707797  KX707798  KX707799 | KX707800  KX707801  KX707802  KX707803 | KX707804  KX707805  KX707806  KX707807 |  |
| **MM104** | KX708445  KX708446  KX708447  KX708448  KX708449  KX708450  KX708451  KX708452 | KX708394  KX708395  KX708396  KX708397  KX708398  KX708399  KX708400  KX708401  KX708402  KX708403  KX708404  KX708405  KX708406  KX708407  KX708408  KX708409  KX708410 | KX708453  KX708454  KX708455  KX708456 | KX708457  KX708458  KX708459 |
| **MM1** | KX708040  KX708041  KX708042  KX708043  KX708044  KX708045  KX708046  KX708047  KX708048  KX708049  KX708050  KX708051  KX708052  KX708053  KX708054  KX708055  KX708056  KX708057  KX708058  KX708059  KX708060  KX708061 | KX708062  KX708063  KX708064 | KX708065  KX708066  KX708067  KX708068  KX708069  KX708070  KX708071 | KX708072  KX708073  KX708074 |
| **MM24** | KX708017  KX708018  KX708019  KX708020  KX708021  KX708022 | KX708023  KX708024  KX708025  KX708026  KX708027  KX708028  KX708029  KX708030  KX708031  KX708032  KX708033 | KX708034  KX708035  KX708036  KX708037 | KX708038  KX708039 |
| **MM12** | KX708176  KX708177  KX708178  KX708179  KX708180 | KX708181  KX708182  KX708183  KX708184 |  | KX708185  KX708186  KX708187 |
| **MM100** | KX708460  KX708461  KX708462  KX708463  KX708464  KX708465  KX708466  KX708467  KX708468  KX708469  KX708470  KX708471  KX708472  KX708473  KX708474  KX708475 | KX708436  KX708437  KX708438  KX708439 | KX708440  KX708441  KX708442 | KX708443  KX708444 |
| **MM117** | KX708228  KX708229  KX708230  KX708231  KX708232  KX708233  KX708234  KX708235  KX708236  KX708237  KX708238  KX708239 | KX708240  KX708241 | KX708242  KX708243  KX708244  KX708245 | KX708246 |
| **MM122** | KX708247  KX708248  KX708249  KX708250  KX708251  KX708252  KX708253  KX708254  KX708255  KX708256  KX708257  KX708258  KX708259  KX708260  KX708261  KX708262  KX708263  KX708264  KX708265  KX708266 | KX708267  KX708268  KX708269  KX708270 | KX708271  KX708272  KX708273 | KX708274  KX708275 |
| **MM99** | KX707827  KX707828  KX707829  KX707830  KX707831  KX707832  KX707833  KX707834  KX707835  KX707836  KX707837  KX707838  KX707839  KX707840  KX707841  KX707842  KX707843  KX707844  KX707845  KX707846  KX707847 | KX707848  KX707849  KX707850  KX707851  KX707852 | KX707853  KX707854  KX707855  KX707856 | KX707857 |
| **MM14** | KX708207  KX708208  KX708209  KX708210  KX708211  KX708212  KX708213  KX708214  KX708215  KX708216  KX708217  KX708218  KX708219 | KX708112  KX708113  KX708114  KX708115 | KX708116  KX708117 | KX708220 |
| **MM105** | KX708411  KX708412 | KX708413  KX708414  KX708415 | KX708416  KX708417  KX708418  KX708419 | KX708420 |
| **MM111** | KX708290  KX708291  KX708292  KX708293  KX708294  KX708295  KX708296  KX708297  KX708298  KX708299  KX708300  KX708301 | KX708302  KX708303  KX708304  KX708305  KX708306  KX708307  KX708308  KX708309  KX708310  KX708311  KX708312  KX708313  KX708314 | KX708315  KX708316 | KX708317  KX708318  KX708319 |
| **MM108** | KX708385  KX708386  KX708387  KX708388  KX708389  KX708390  KX708391  KX708392  KX708393 |  | KX708331  KX708332  KX708333  KX708334 | KX708335  KX708336 |
| **MM106** | KX708421  KX708422  KX708423  KX708424  KX708425  KX708426  KX708427  KX708428  KX708429  KX708430  KX708431  KX708432  KX708433 | KX708357  KX708358  KX708359  KX708360  KX708361  KX708362 | KX708434  KX708435 |  |
| **MM128** | KX708276  KX708277  KX708278  KX708279  KX708280  KX708281  KX708282  KX708283  KX708284 | KX708221  KX708222  KX708223  KX708224  KX708225  KX708226 |  | KX708227 |
| **MM10** | KX708320  KX708321  KX708322  KX708323  KX708324  KX708325  KX708326  KX708327  KX708328  KX708329 | KX708285  KX708286  KX708287 | KX708288  KX708289 | KX708330 |
| **MM13** | KX708188  KX708189  KX708190  KX708191  KX708192  KX708193 | KX708194  KX708195  KX708196  KX708197  KX708198  KX708199  KX708200  KX708201  KX708202  KX708203  KX708204 | KX708205  KX708206 |  |
| **MM68** | KX707899  KX707900  KX707901  KX707902 | KX707903  KX707904  KX707905 | KX707906  KX707907  KX707908  KX707909 | KX707910  KX707911 |
| **MM59** | KX707882  KX707883  KX707884  KX707885  KX707886  KX707887  KX707888  KX707889 | KX707890  KX707891  KX707892  KX707893 | KX707894  KX707895  KX707896  KX707897 | KX707898 |
| **MM5** | KX707858  KX707859  KX707860  KX707861  KX707862  KX707863  KX707864  KX707865  KX707866  KX707867  KX707868 | KX707869  KX707870  KX707871  KX707872  KX707873  KX707874  KX707875 | KX707876  KX707877  KX707878  KX707879  KX707880  KX707881 |  |
| **MM107** | KX708363  KX708364  KX708365  KX708366  KX708367  KX708368  KX708369  KX708370  KX708371  KX708372  KX708373  KX708374  KX708375  KX708376 | KX708377  KX708378  KX708379  KX708380 | KX708381  KX708382  KX708383  KX708384 |  |
| **MM109** | KX708337  KX708338  KX708339  KX708340  KX708341  KX708342  KX708343  KX708344  KX708345  KX708346  KX708347 | KX708348  KX708349  KX708350  KX708351 | KX708352  KX708353  KX708354  KX708355 | KX708356 |
| **MM7** | KX707808  KX707809  KX707810  KX707811  KX707812  KX707813  KX707814  KX707815  KX707816  KX707817 | KX707818  KX707819  KX707820  KX707821  KX707822 | KX707823  KX707824  KX707825  KX707826 |  |
| **MM19** | KX708163  KX708164  KX708165  KX708166 | KX708167  KX708168  KX708169  KX708170  KX708171 | KX708172  KX708173  KX708174  KX708175 |  |
| **MM44** | KX707927  KX707928  KX707929  KX707930  KX707931  KX707932  KX707933  KX707934 | KX707935  KX707936  KX707937  KX707938  KX707939  KX707940  KX707941  KX707942  KX707943  KX707944  KX707945  KX707946  KX707947  KX707948  KX707949  KX707950  KX707951  KX707952  KX707953  KX707954  KX707955  KX707956  KX707957  KX707958 | KX707959  KX707960  KX707961  KX707962 |  |
| **MM21** | KX708075  KX708076  KX708077  KX708078  KX708079  KX708080  KX708081  KX708082  KX708083 | KX708084  KX708085  KX708086  KX708087  KX708088  KX708089  KX708090  KX708091  KX708092  KX708093  KX708094  KX708095  KX708096  KX708097  KX708098  KX708099  KX708100  KX708101  KX708102  KX708103  KX708104  KX708105  KX708106  KX708107 | KX708108  KX708109  KX708110  KX708111 |  |
| **MM23** | KX707989  KX707990  KX707991  KX707992  KX707993 | KX707994  KX707995  KX707996  KX707997  KX707998  KX707999  KX708000  KX708001  KX708002  KX708003  KX708004  KX708005  KX708006  KX708007  KX708008  KX708009  KX708010  KX708011 | KX708012  KX708013  KX708014  KX708015 | KX708016 |
| **MM3** | KX707976  KX707977  KX707978  KX707979  KX707980  KX707981  KX707982 | KX707912  KX707913  KX707914  KX707915  KX707916  KX707917  KX707918  KX707919  KX707920  KX707921  KX707922  KX707923  KX707924  KX707925  KX707926 | KX707983  KX707984  KX707985  KX707986  KX707987  KX707988 |  |
