# Supplementary figures and images for: A Phenotypic and Genotypic Analysis of the Antimicrobial Potential of Cultivable Streptomyces Isolated from Cave Moonmilk Deposits
Source: Front Microbiol. 2016 Sep 21;7:1455. doi: 10.3389/fmicb.2016.01455 (PMC5030222; doi:10.3389/fmicb.2016.01455)

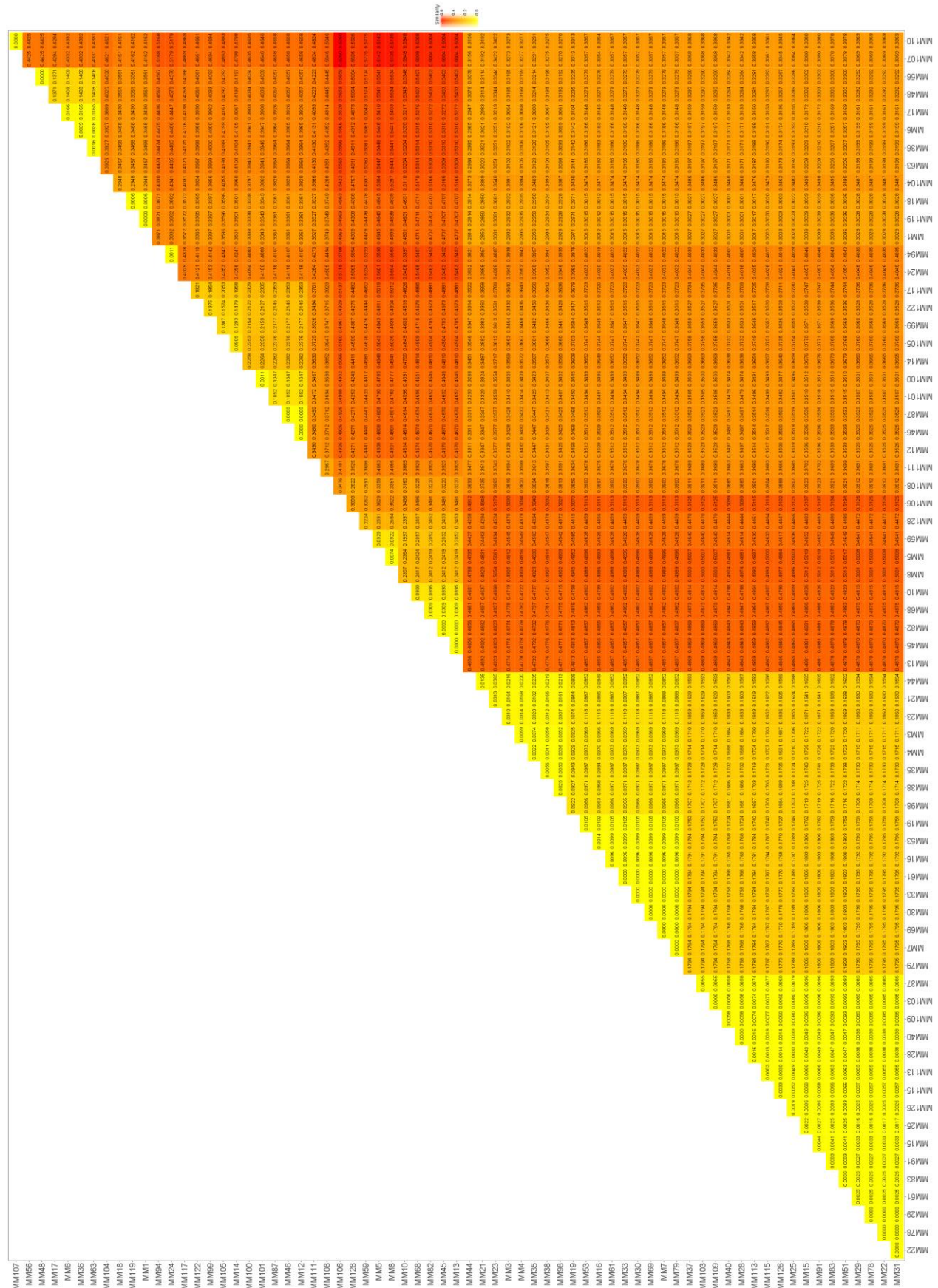

Supplement: Supplementary file 5 [file Image_1.PDF]
