# Supplementary material for: A Phenotypic and Genotypic Analysis of the Antimicrobial Potential of Cultivable Streptomyces Isolated from Cave Moonmilk Deposits
Source: Front Microbiol. 2016 Sep 21;7:1455. doi: 10.3389/fmicb.2016.01455 (PMC5030222; doi:10.3389/fmicb.2016.01455)

**Supplementary Figure 2.** Mass spectra comparison of the antifungal metabolite synthetized by MM99 (top spectra) with the reference cycloheximide (C1988, Sigma-Aldrich) compound (bottom spectra).

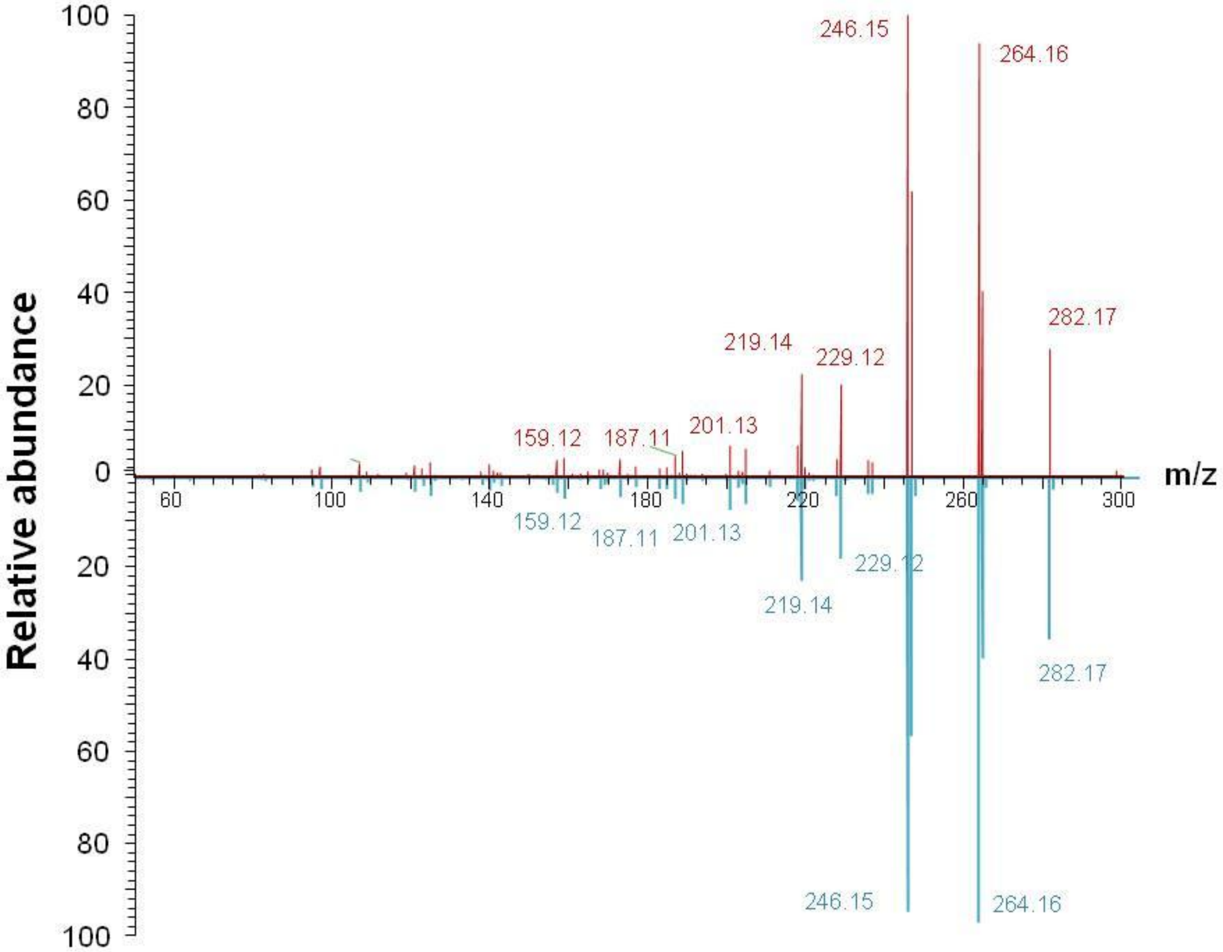

Supplement: Supplementary file 6 [file Image_2.PDF]
